# Cardiac mortality after radiotherapy, chemotherapy and endocrine therapy for breast cancer: Cohort study of 2 million women from 57 cancer registries in 22 countries

**DOI:** 10.1002/ijc.32908

**Published:** 2020-03-04

**Authors:** Katherine E. Henson, Paul McGale, Sarah C. Darby, Max Parkin, Yaochen Wang, Carolyn W. Taylor

**Affiliations:** ^1^ Nuffield Department of Population Health University of Oxford Oxford United Kingdom; ^2^ National Cancer Registration and Analysis Service, Public Health England London United Kingdom

**Keywords:** breast cancer, heart disease, radiotherapy

## Abstract

Comparisons of patients receiving different cancer treatments reflect the effects of both treatment and patient selection. In breast cancer, however, if radiotherapy decisions are unrelated to laterality, comparisons of left‐sided and right‐sided cancers can demonstrate the causal effects of higher‐*versus*‐lower cardiac radiation dose. Cardiac mortality was analysed using individual patient data for 1,934,248 women with breast cancer in 22 countries. The median date of diagnosis was 1996 and the interquartile range was 1987–2002. A total of 1,018,505 women were recorded as irradiated, 223,077 as receiving chemotherapy, 317,619 as receiving endocrine therapy and 55,264 died of cardiac disease. Analyses were stratified by time since breast cancer diagnosis, age at diagnosis, calendar year of diagnosis and country. Patient‐selection effects were evident for all three treatments. For radiotherapy, there was also evidence of selection according to laterality in women irradiated 1990 or later. In patients irradiated before 1990, there was no such selection and cardiac mortality was higher in left‐sided than right‐sided cancer (rate ratio [RR]: 1.13, 95% confidence interval 1.09–1.17). Left‐*versus*‐right cardiac mortality RRs were greater among younger women (1.46, 1.19, 1.20, 1.09 and 1.08 after cancer diagnoses at ages <40, 40–49, 50–59, 60–69 and 70+ years, 2*p*_trend_ =0.003). Left‐*versus*‐right RRs also increased with time since cancer diagnosis (1.03, 1.11, 1.19 and 1.21 during 0–4, 5–14, 15–24 and 25+ years, 2*p*_trend_ =0.002) while for women who also received chemotherapy, the left‐*versus*‐right RR was 1.42 (95% confidence interval 1.13–1.77), compared to 1.10 (1.05–1.16) for women who did not (2*p*_difference_= 0.03). These results show that the relative increase in cardiac mortality from cardiac exposure during breast cancer radiotherapy given in the past was greater in younger women, lasted into the third decade after exposure and was greater when chemotherapy was also given.

## Abstract 2

**What's new?**

Cardiac mortality was examined using individual patient data for 1,934,248 women with breast cancer in 22 countries. Patient‐selection effects dominated most treatment comparisons. However, comparison of left‐sided versus right‐sided cancers in women recorded as irradiated before 1990 provided evidence of a causal effect of higher heart radiation dose on cardiac mortality. The relative effect was greater at younger ages, lasted over 25 years, and was greater in women who also received chemotherapy.

## Introduction

Randomised trials have shown that radiotherapy, chemotherapy and endocrine therapy for early‐stage breast cancer can reduce the rates of recurrence and of breast cancer death.^1^, ^2^, ^3^, ^4^ However, long‐term follow‐up of some trials has also shown that radiotherapy and chemotherapy can increase the risk of heart disease,^5^, ^6^ thereby reducing the extent to which overall survival is improved by these treatments and reducing quality of life for some breast cancer survivors. In contrast, there is evidence that tamoxifen may reduce the risk of heart disease.^5^, ^7^

Observational epidemiological studies comparing heart disease rates in breast cancer patients selected to receive different cancer treatments in the general population are of interest but must be interpreted with caution.^8^ This is because more aggressive treatments may be selectively prescribed for patients with adverse disease characteristics or favourable comorbidity profiles. Larger proportions of younger patients are likely to have adverse disease characteristics and also to have favourable comorbidity profiles, so any selection effects may be associated with the age at which a woman is diagnosed with breast cancer.

For the particular case of radiotherapy, it may be possible to draw stronger conclusions from observational data than for chemotherapy or endocrine therapy because cardiac radiation doses are usually larger in left‐sided than in right‐sided breast cancer. Therefore studies comparing heart disease rates in women irradiated for left‐sided and right‐sided breast cancer may reveal the effects of higher *versus* lower cardiac radiation doses.^9^ While some such studies, especially those published more recently, have found no significant increase in heart disease in women with left‐sided breast cancer compared to right‐sided,^10^, ^11^, ^12^ others are consistent with randomised radiotherapy trials and have provided additional insights into radiation‐related cardiac risks.^13^ When combined with detailed information about cardiac radiation doses received by individual women, studies comparing women with left‐sided and right‐sided breast cancer have also shown that the dose–response relationship for subsequent risk of ischaemic heart disease is approximately linear with no apparent threshold.^14^, ^15^ In addition, they have demonstrated that, for a given dose of radiation to the heart, the absolute radiation‐related risk of ischaemic heart disease is greater in women with pre‐existing cardiac risk factors.^14^

Comparisons of heart disease rates in women irradiated for left‐sided *versus* right‐sided breast cancer can, however, only show the causal effect of higher *versus* lower cardiac radiation dose if the decision to give radiotherapy is unrelated to cancer laterality. This may have been the case prior to 1990 because, until then, it was widely thought that the heart was resistant to radiation. However, in 1990, a study was published showing that patients with left‐sided breast cancer had higher subsequent mortality from myocardial infarction than patients with right‐sided breast cancer.^16^ Since then, oncologists have become increasingly aware of the cardiac risks of radiotherapy and so they may be less likely to give radiotherapy in women who already have heart disease if the cancer is in the left rather than the right breast.

Information on large numbers of women with breast cancer treated both before and after 1990 is necessary to examine the evidence regarding the selection of women for different adjuvant treatments, and whether any such selection is related to age at cancer diagnosis or the laterality of the cancer. We therefore collated individual patient information from cancer registries and other organisations round the world to provide further insight on adjuvant treatments for breast cancer and subsequent heart disease.

## Materials and Methods

Voting members of the International Association of Cancer Registries^17^ in high income countries^18^ and other relevant organisations were approached to identify those who held information on women diagnosed with breast cancer and who had followed them prospectively, recording date and cause of death for women who died. Those who could also provide information on breast cancer laterality and use of radiotherapy were invited to contribute data, and all accepted (Supporting Information Table S1, Text S1).

In the data contributed by each registry, individual women were included if they had been diagnosed with invasive breast cancer or with ductal carcinoma *in situ* (DCIS) and had known disease laterality. Exclusion criteria were: male sex, bilateral breast cancer/carcinoma *in situ*, previous invasive cancer (apart from nonmelanoma skin cancer), emigration or immigration at any time, death on recorded date of breast cancer/carcinoma *in situ* diagnosis or previous thoracic radiotherapy. For women who had died, the cause of death was taken to be the certified underlying cause. The codes used to define cardiac disease are given in Supporting Information Table S2. The data contributed by each organisation are listed in Supporting Information Table S3. Ethical approval for the study was obtained from the South Central Research Ethics Committee of the UK National Health Service Health Research Authority. Our study comprises statistical analysis conducted on routinely collected data that had been depersonalised. Individual consent was therefore deemed unnecessary.

According to the analysis plan, agreed by contributing registries, each woman's contribution to the person‐years at risk began from her date of breast cancer diagnosis and ended on her date of death, loss to follow‐up, 85th birthday or the date she was last known to be alive—whichever was earliest. Mortality rate ratios (RR) were estimated by Poisson regression with stratification by time since breast cancer diagnosis (0, 5‐9, 10‐14, 15‐19, 20‐24, 25‐30, 30+ years), age at breast cancer diagnosis (<20, 20‐24, 25‐29, 30‐34, 35‐39, 40‐44, 45‐49, 50‐54, 55‐59, 60‐64, 65‐69, 70‐74, 75+ years), calendar year of breast cancer diagnosis (<1950, 1950‐1954, 1955‐1959, 1960‐1964, 1965‐1969, 1970‐1974, 1975‐1979, 1980‐1984, 1985‐1989, 1990‐1994, 1995‐1999, 2000‐2004, 2005+) and country. Significance tests used the likelihood ratio and were two‐sided, and confidence intervals were based on standard errors. No adjustments to *p* values for multiple testing were made. This is because the comparison of women with left‐sided and right‐sided breast cancer was an *a priori* hypothesis. Other comparisons were to some extent data driven. Calculations were performed using Stata version 15.

Registries and other participating organisations depersonalised their data and transferred them to Oxford where they were collated and analysed. Representatives from each organisation contributing data were invited to a meeting, where data summaries and provisional analyses were discussed. These were modified in the light of comments received. Comparisons of mortality from heart disease in women with left‐sided and right‐sided breast cancer had previously been published for several participating registries and other organisations individually, including several hundred thousand individuals in total.^10^, ^11^, ^12^, ^16^, ^19^, ^20^, ^21^, ^22^, ^23^, ^24^, ^25^, ^26^, ^27^, ^28^, ^29^, ^30^, ^31^, ^32^, ^33^, ^34^, ^35^, ^36^, ^37^ However, bringing the data together in the present study, provides much greater power to detect differences in subgroups of interest.

### Data availability

The data presented in this paper remain the property of the contributing registries and organisations. Oxford University's policies for accessing them are available: https://www.ndph.ox.ac.uk/about/data-access.

## Results

### Characteristics of the study population

A total of 1,934,248 women from 57 cancer registries and other organisations in 22 countries were included in the study, of which 996,285 had left breast cancer and 937,963 had right breast cancer (Tables 1 and 2). The median date of diagnosis was 1996 and the interquartile range was 1987–2002. The mean age at breast cancer diagnosis was 57 years and the mean length of follow‐up was 6.7 years. A total of 1,018,505 women (52.7%) were recorded as receiving radiotherapy, 223,077 (11.5%) as receiving chemotherapy and 317,619 (16.4%) as receiving endocrine therapy. A total of 55,264 deaths from cardiac disease were reported.

**Table 1:** Characteristics of women in the study and numbers of deaths attributed to cardiac disease

	Women		Cardiac deaths	
	Number	Percentage	Number	Percentage
Calendar year of cancer diagnosis				
<1970	92,662	4.8	6,806	12.3
1970–1979	167,531	8.7	10,800	19.5
1980–1989	341,040	17.6	16,506	29.9
1990–1999	615,799	31.8	15,672	28.4
2000+	717,216	37.1	5,480	9.9
Age at cancer diagnosis (years)				
<40	133,657	6.9	632	1.1
40–49	399,512	20.7	3,674	6.6
50–59	519,969	26.9	8,623	15.6
60–69	505,643	26.1	18,664	33.8
70–79	375,467	19.4	23,671	42.8
Race/ethnicity				
White	915,995	47.4	33,136	60.0
Black	66,850	3.5	2,690	4.9
Asian/Pacific	58,534	3.0	1,009	1.8
Other/Unknown^1^	273,260	14.1	4,625	8.4
Not recorded^2^	619,609	32.0	13,804	25.0
Stage at cancer diagnosis				
DCIS	131,774	6.8	2,138	3.9
Localised	738,340	38.2	24,625	44.6
Regional	474,194	24.5	13,784	24.9
Unknown/Metastatic	388,352	20.1	7,397	13.4
Not recorded^2^	201,588	10.4	7,320	13.2
Surgery				
BCS	515,111	26.6	9,637	17.4
Mastectomy	485,809	25.1	17,042	30.8
Unknown/None	769,874	39.8	22,190	40.2
Not recorded^2^	163,454	8.5	6,395	11.6
Radiotherapy				
Yes	1,018,505	52.7	22,100	40.0
No	915,743	47.3	33,164	60.0
Chemotherapy				
Yes	223,077	11.5	1,368	2.5
No	685,502	35.4	18,536	33.5
Unknown	31,453	1.6	399	0.7
Not recorded^2^	994,216	51.4	34,961	63.3
Endocrine therapy				
Yes	317,619	16.4	5,643	10.2
No	558,145	28.9	14,156	25.6
Unknown	15,482	0.8	277	0.5
Not recorded^2^	1,043,002	53.9	35,188	63.7
Region				
Europe: Britain and Ireland				
Britain	343,984	17.8	6,185	11.2
Ireland	13,594	0.7	114	0.2
Europe: Nordic countries				
Denmark	101,880	5.3	5,678	10.3
Finland	76,194	3.9	5,310	9.6
Norway	20,310	1.1	187	0.3
Sweden	48,060	2.5	1,753	3.2
Europe: Other countries				
Austria	11,608	0.6	223	0.4
Estonia	11,043	0.6	722	1.3
France	6,017	0.3	192	0.3
Germany	236,846	12.2	2,283	4.1
Italy	39,704	2.1	555	1.0
Lithuania	10,812	0.6	192	0.3
Netherlands	25,420	1.3	235	0.4
Slovakia	28,139	1.5	73	0.1
Slovenia	8,863	0.5	135	0.2
Spain	5,855	0.3	57	0.1
Switzerland	6,976	0.4	105	0.2
North America				
Canada	106,111	5.5	2,311	4.2
USA	778,048	40.2	27,929	50.5
Other regions				
Australia	46,993	2.4	845	1.5
Israel	4,827	0.2	108	0.2
Japan	2,964	0.2	72	0.1
All women	1,934,248	100.0	55,264	100.0

Data contributed by individual registries is listed in Supporting Information Table S3.

1 Variable recorded by registry but unknown for some individuals.

2 Variable not recorded by registry.

Abbreviations: BCS, breast‐conserving surgery; DCIS, ductal carcinoma *in situ*.

**Table 2:** Left‐sided and right‐sided breast cancer, numbers of women and percentages irradiated

	Number of women		Percentage irradiated			*p*‐value
	Left	Right	Left	Right	Difference	
*Women diagnosed before 1990 (Mean follow‐up 8.9 years)*						
Age at cancer diagnosis (years)						
<60	164,208	154,272	53.02	52.91	0.11	0.54
60+	147,154	135,599	44.81	44.85	−0.04	0.81
All ages	311,362	289,871	49.14	49.14	0.00	0.98
*Women diagnosed 1990+ (Mean follow‐up 5.8 years)*						
Age at cancer diagnosis (years)						
<60	375,479	359,179	56.95	57.11	−0.16	0.16
60+	309,444	288,913	50.66	51.01	−0.35	0.007
All ages	684,923	648,092	54.10	54.39	−0.29	0.001
*All women*	996,285	937,963	52.55	52.77	−0.22	0.003

For a more detailed tabulation see Supporting Information Table S5.

### Cardiac mortality in treated *versus* untreated women

**Figure 1:**
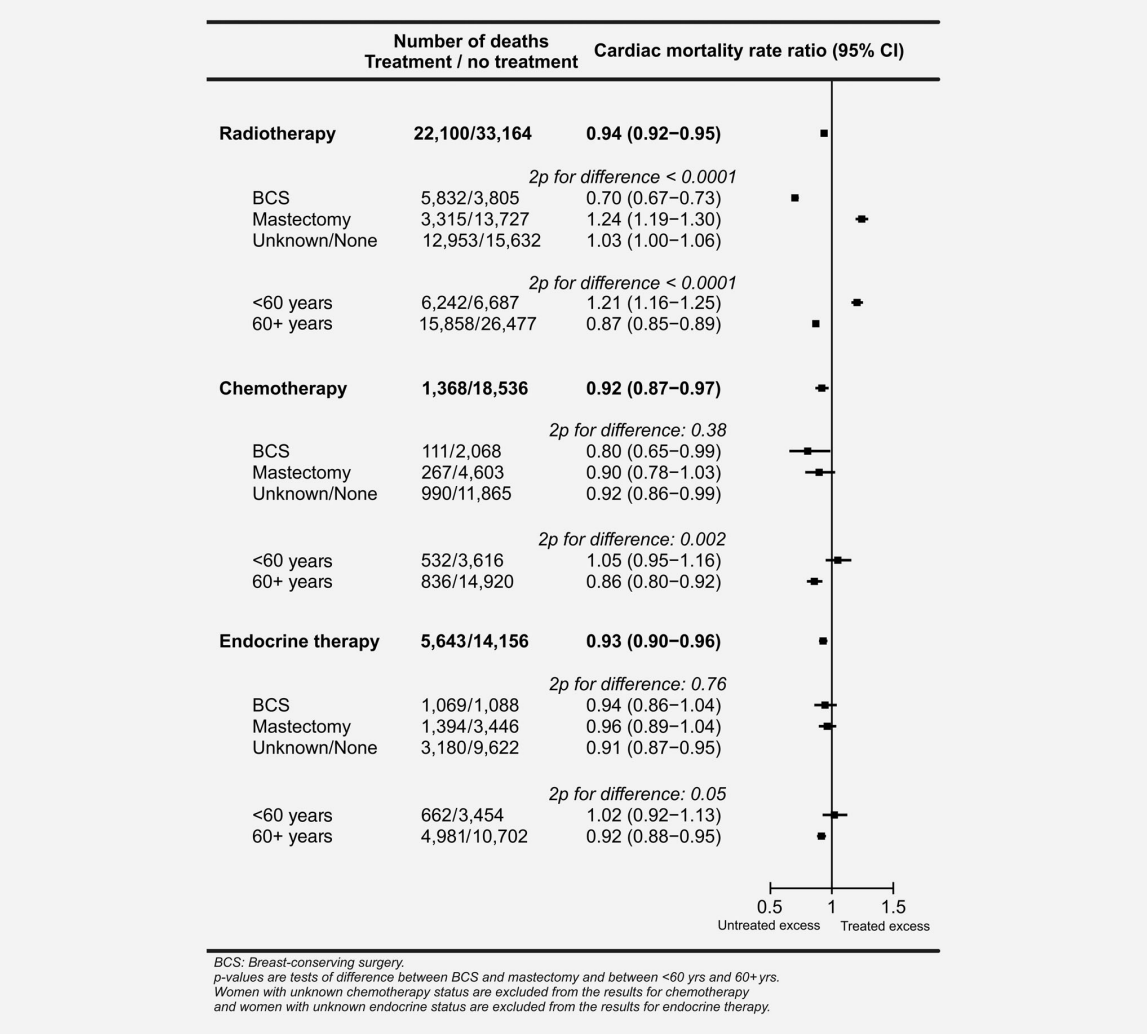
Treated *versus* untreated women: Cardiac mortality rate ratios for women recorded as receiving radiotherapy, chemotherapy or endocrine therapy, overall and by type of surgery and age at breast cancer diagnosis. Rate ratios estimated by Poisson regression with stratification by time since breast cancer diagnosis, age at breast cancer diagnosis, calendar year of breast cancer diagnosis and country.

When all women were considered together, cardiac mortality in women recorded as receiving radiotherapy was lower than in those reported as not receiving it (cardiac mortality RR, irradiated *versus* not, 0.94, 95% confidence interval [CI] 0.92–0.95; Fig. 1). Further analyses showed, however, that radiotherapy given after breast‐conserving surgery (BCS) was associated with a substantial deficit in cardiac mortality (RR, irradiated *versus* not, 0.70, 95% CI 0.67–0.73) while, in contrast, radiotherapy after mastectomy was associated with a substantial excess in cardiac mortality (RR, irradiated *versus* not, 1.24, 95% CI 1.19–1.30, 2*p*_difference_ < 0.0001). This pattern was present not only overall, but throughout Europe, in North America and elsewhere (Supporting Information Fig. S1). Analyses subdividing women by age at radiotherapy showed also that radiotherapy given to younger women (<60 years) was associated with a substantial excess of cardiac mortality (RR, irradiated *versus* not, 1.21, 95% CI 1.16–1.25), whereas radiotherapy given to older women was associated with a considerable deficit (RR, irradiated *versus* not, 0.87, 96% CI 0.85–0.89, 2*p*_difference_ < 0.0001).

When women reported as receiving chemotherapy were compared to women reported as not receiving it, chemotherapy use was associated with a deficit in cardiac mortality overall (RR, chemotherapy *versus* not, 0.92, 95% CI 0.87–0.97). The same was true for endocrine therapy (RR, endocrine therapy *versus* not, 0.93, 95% CI 0.90–0.96). In neither case was there any evidence of a difference between women given BCS and women given mastectomy (chemotherapy 2*p*_difference_ = 0.38, endocrine therapy 2p_difference_ = 0.76). However, for both these systemic treatments, the deficit was present only in older women (RR chemotherapy *versus* not, <60 years: 1.05, 95% CI 0.95–1.16, in women, 60+ years: 0.86, 95% CI 0.80–0.92, 2*p*_difference_= 0.002; RR endocrine therapy *versus* not, <60 years: 1.02, 95% CI 0.92–1.13, in women 60+ years: 0.92, 95% CI 0.88–0.95, 2*p*_difference_=0.05).

Further analyses showed that, for each of these three treatments, lower mortality remained in women aged 60+ who received the treatment compared to women who did not, even after taking into account the other two treatments (Supporting Information Table S4).

### Percentages of women recorded as irradiated

The percentages of women reported as receiving radiotherapy varied substantially with some factors. For example, in some countries more than 70% of women were reported as receiving it compared to less than 40% in others, while over 70% of women who had BCS received radiotherapy compared to fewer than 30% of women who had mastectomy (Supporting Information Tables S3 and S5).

As expected, differences between the percentages of women recorded as receiving radiotherapy in left‐sided and right‐sided breast cancer were much smaller. There was, however, a significant difference between the two (left‐sided 52.55%, right‐sided 52.77%, percentage difference −0.22, 2*p*_difference=_ 0.003; Table 2). This difference varied with calendar year of diagnosis and with age at diagnosis. For women diagnosed prior to 1990, the percentage irradiated was identical in left‐sided and right‐sided breast cancer for all ages at cancer diagnosis combined (49.14%) and when women aged <60 and 60+ years were considered separately the percentage differences in this <1990 group were also small and did not reach statistical significance. In contrast, from 1990 onwards, fewer women with left‐sided than right‐sided breast cancer received radiotherapy (left‐sided 54.10%, right‐sided 54.39%, percentage difference −0.29, 2*p*_difference_= 0.001) and the difference was principally in women aged 60+ years at diagnosis (left‐sided 50.66%, right‐sided 51.01%, percentage difference −0.35, 2*p*_difference_ =0.007) while in women aged <60 at cancer diagnosis the difference was small and did not reach statistical significance (left‐sided 56.95%, right‐sided 57.11%, percentage difference −0.16, 2*p*_difference=_ 0.16). Therefore, as there was evidence of patient selection according to laterality and age in women diagnosed 1990+, but not in women diagnosed <1990, many of our subsequent analyses considered these groups separately.

### Cardiac mortality in left‐sided *versus* right‐sided breast cancer

**Figure 2:**
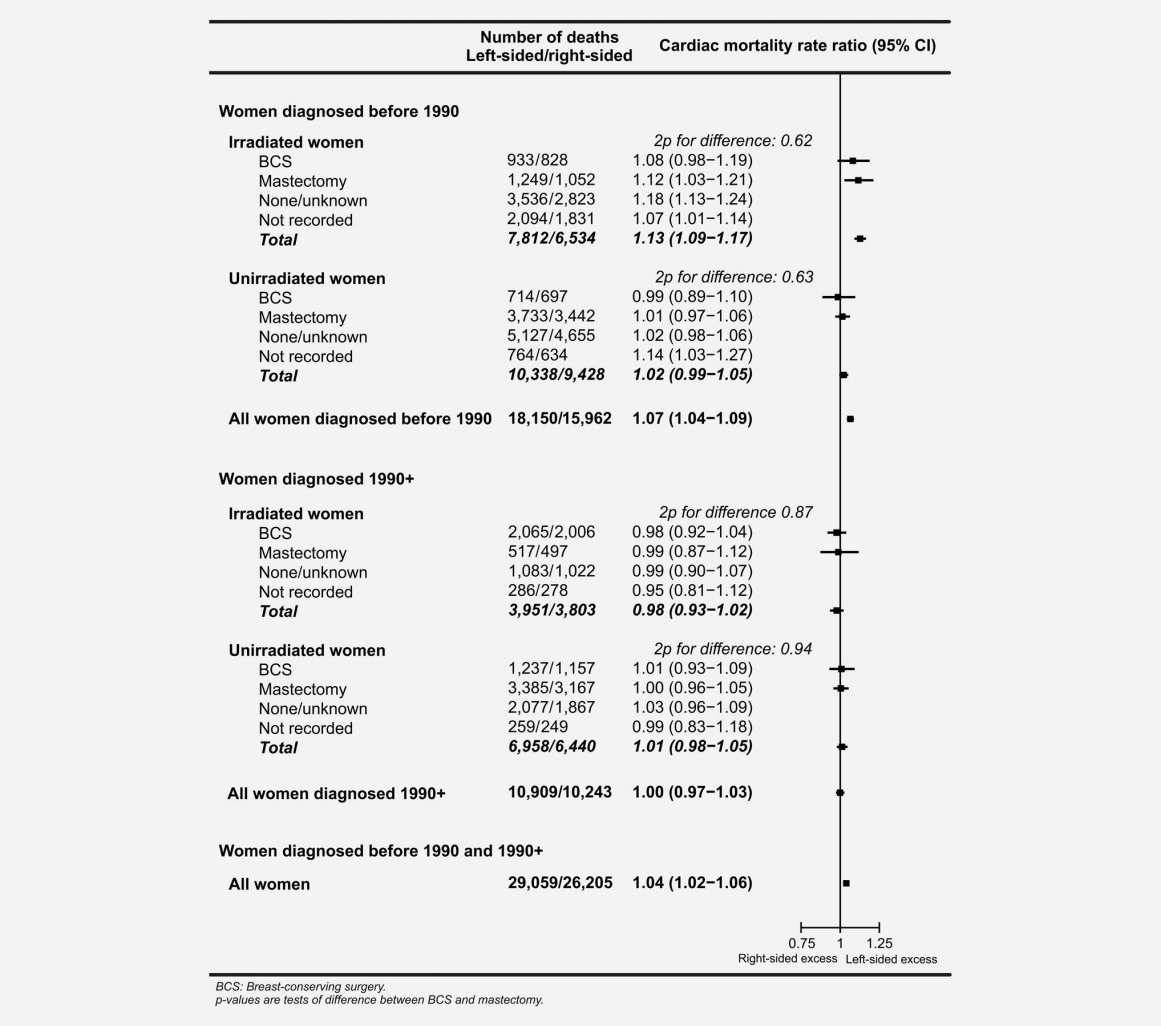
Women with left‐sided breast cancer *versus* women with right‐sided breast cancer: Cardiac mortality rate ratios by calendar period, whether or not they were recorded as irradiated and type of surgery. Rate ratios estimated by Poisson regression with stratification by time since breast cancer diagnosis, age at breast cancer diagnosis, calendar year of breast cancer diagnosis and country. The subtotals and overall total are also stratified by radiotherapy.

When all women were considered together, cardiac mortality was higher in women with left‐sided cancer than in women with right‐sided cancer (RR, left‐*versus*‐right, 1.04, 95% CI 1.02–1.06; Fig. 2). The increase was, however, confined to women whose cancer was diagnosed before 1990 and who were recorded as receiving radiotherapy (RR, left‐*versus*‐right, irradiated 1.13, 95% CI 1.09–1.17). During this period, there was little evidence of an increase in the absence of radiotherapy (RR, left‐*versus*‐right, unirradiated 1.02 95% CI 0.99–1.05, 2*p*_difference_ between irradiated and unirradiated: <0.0001). In neither of these groups did the RR, left‐*versus*‐right, differ significantly between women given BCS and women given mastectomy (irradiated RR left‐*versus*‐right: BCS 1.08, 95% CI 0.98–1.19, Mastectomy 1.12, 95% CI 1.03–1.21, 2*p*_difference_= 0.62; unirradiated RR left‐*versus*‐right: BCS 0.99, 95%CI 0.89–1.10, Mastectomy 1.01, 95% CI 0.97–1.06, 2*p*_difference_= 0.63).

Considering all women whose cancer was diagnosed during 1990+, the cardiac mortality RR left‐*versus*‐right was 1.00 (0.97–1.03; Fig. 2) and the RRs left‐*versus*‐right were close to unity in both women recorded as irradiated and women recorded as unirradiated, regardless of the type of surgery they had received.

### Cardiac mortality, left‐sided *versus* right‐sided breast cancer, in women recorded as receiving radiotherapy

**Figure 3:**
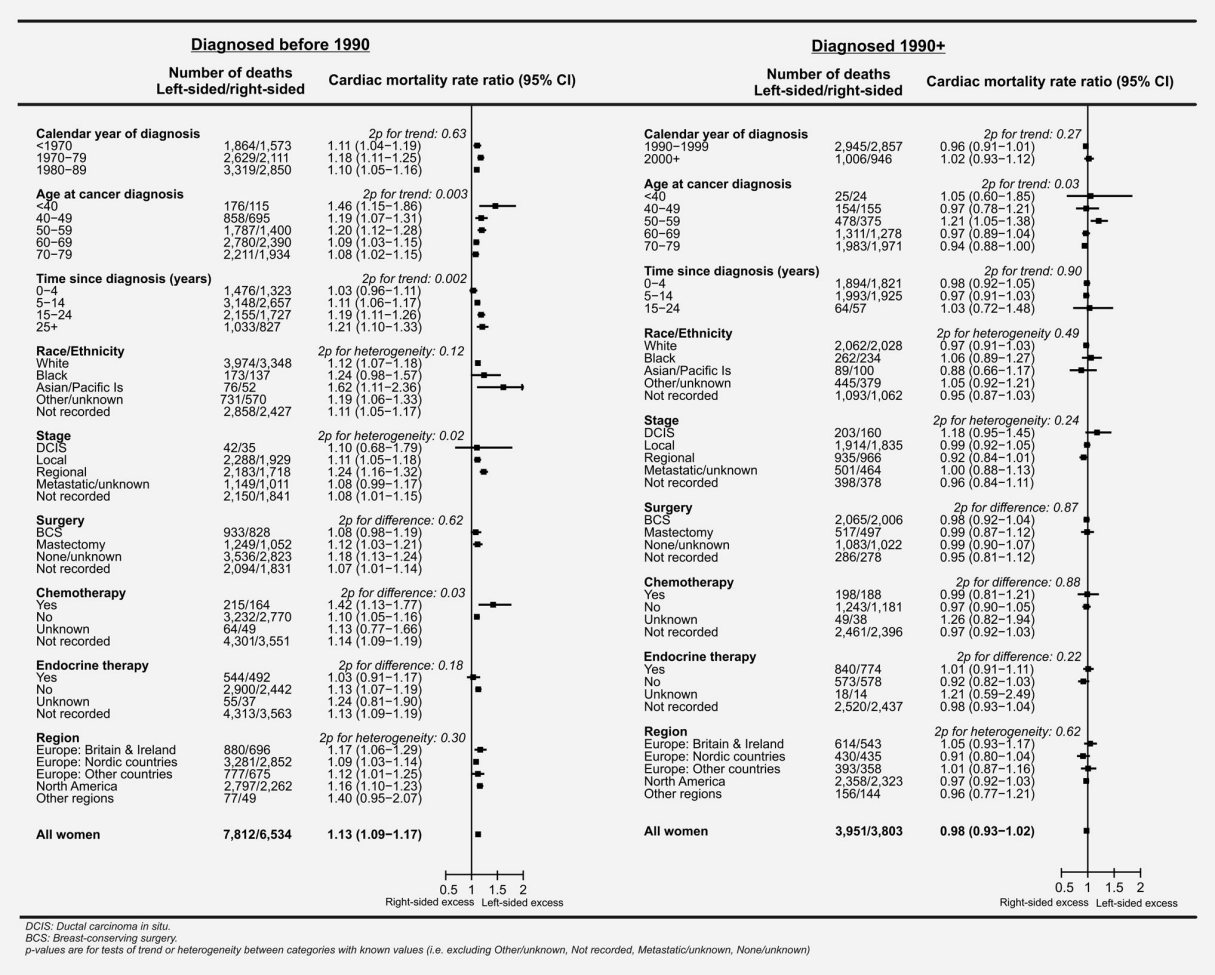
Women recorded as irradiated with left‐sided breast cancer *versus* women recorded as irradiated with right‐sided breast cancer: Cardiac mortality rate ratios by patient, tumour and treatment characteristics and geographic region. Rate ratios estimated by Poisson regression with stratification by time since breast cancer diagnosis, age at breast cancer diagnosis, calendar year of breast cancer diagnosis and country. See Supporting Information Figure S2 for analyses of women irradiated <1990 and 1990+ combined.

Considering all women diagnosed before 1990 and recorded as receiving radiotherapy, there was no evidence of a trend in the cardiac mortality RR, left‐*versus*‐right, with calendar year of cancer diagnosis (2*p*_trend=_ 0.63; Fig. 3, left‐hand panel). The cardiac mortality RRs left‐*versus*‐right were higher in women who were younger at cancer diagnosis (cardiac mortality RRs left‐*versus*‐right: 1.46, 1.19, 1.20, 1.09 and 1.08 at ages <40, 40–49, 50–59, 60–69 and 70–79 years, respectively, 2*p*_trend_= 0.003) and they increased with increasing time since cancer diagnosis (cardiac mortality RRs left‐*versus*‐right 1.03, 1.11, 1.19 and 1.21 for 0–4, 5–14, 15–24 and 25+ years, 2*p*_trend=_ 0.002). Larger left‐*versus*‐right RRs were also observed in women whose breast cancer involved the regional lymph nodes compared to women whose breast cancer did not (RR left‐*versus*‐right: DCIS 1.10, local 1.11, regional 1.24; 2*p*_heterogeneity=_ 0.02) and in women recorded as receiving chemotherapy (RR left‐*versus*‐right: with chemotherapy 1.42, no chemotherapy 1.10; 2*p*_difference_ =0.03). There was no significant variation according to race/ethnicity (2*p*_heterogeneity_ =0.12), type of surgery (2*p*_difference_ = 0.62), receipt of endocrine therapy (2*p*_difference_= 0.18) or geographical region (2*p*_heterogeneity_ =0.30). Neither was there any significant variation between all countries considered individually (2*p*_heterogeneity_ =0.71) (Supporting Information Figs. S3 and S4).

**Figure 4:**
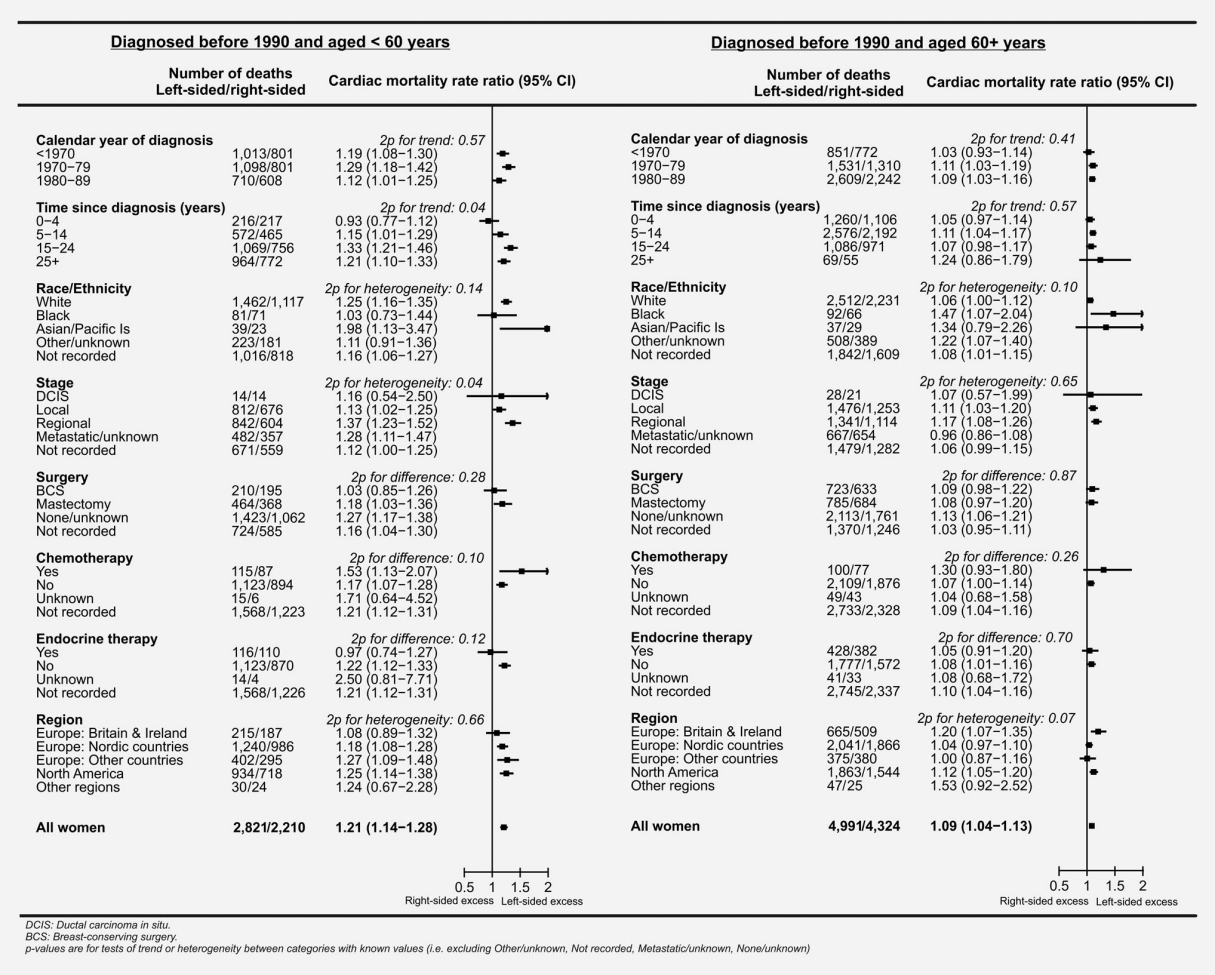
Women recorded as irradiated with left‐sided breast cancer *versus* women recorded as irradiated with right‐sided breast cancer who were diagnosed with breast cancer before 1990: Cardiac mortality rate ratios by age at diagnosis of breast cancer and by other patient, tumour and treatment characteristics and geographic region. Rate ratios estimated by Poisson regression with stratification by time since breast cancer diagnosis, age at breast cancer diagnosis, calendar year of breast cancer diagnosis and country. See Supporting Information Figure S5 for corresponding analyses in women diagnosed 1990+.

The analysis for all women diagnosed before 1990 and recorded as receiving radiotherapy was repeated separately for women aged <60 and 60+ years. For all irradiated women aged <60 at cancer diagnosis, the RR, left‐*versus*‐right for all cardiac mortality was 1.21 (95% CI 1.14–1.28; Fig. 4, left‐hand panel). As for all women who were diagnosed before 1990 and recorded as irradiated, there was no significant trend in the RR, left‐*versus*‐right according to calendar year of cancer diagnosis (RRs 1.19, 1.29 and 1.12 for women diagnosed in periods <1970, 1970–1979 and 1980–1989, 2*p*_trend_ =0.57) while the RR, left‐*versus*‐right increased with increasing time since cancer diagnosis (RRs, left‐*versus*‐right: 0.93, 1.15, 1.33 and 1.21 in periods 0–4, 5–14, 15–24 and 25+ years, 2*p*_trend_ =0.04) and varied with stage of breast cancer (RRs, left‐*versus*‐right: 1.16, 1.13, 1.37 for DCIS, local, regional, respectively, 2*p*_difference_= 0.04). For women recorded as receiving chemotherapy the RR left‐*versus*‐right remained higher than that for women recorded as not receiving it, but with the smaller numbers the difference was no longer statistically significant (with chemotherapy 1.53, without chemotherapy 1.17 2*p*_difference_ =0.10).

For women diagnosed before 1990 when aged 60+ years and recorded as receiving radiotherapy, the RR, left‐*versus*‐right, for all cardiac mortality was raised, but by a smaller relative amount than for women diagnosed with cancer at a younger age (1.09 *versus* 1.21, 2*p*_difference_= 0.003, Fig. 4). In these older women, there was no significant variation in the cardiac mortality RRs left‐*versus*‐right according to any of the factors considered (Fig. 4 right panel).

For women diagnosed during the period 1990+ and recorded as receiving radiotherapy, the RR left‐*versus*‐right for all cardiac mortality was 0.98 (95% CI 0.93–1.02). The cardiac mortality RRs left‐*versus*‐right tended to be slightly higher in women who were younger at cancer diagnosis (cardiac mortality RRs left‐*versus*‐right: 1.05, 0.97, 1.21, 0.97 and 0.94 at ages <40, 40–49, 50–59, 60–69 and 70–79 years, respectively, 2*p*_trend_ =0.03) but did not vary significantly according to any of the other factors considered (Fig. 3 right panel).

### Mortality from different types of heart disease in women recorded as receiving radiotherapy

Among women diagnosed before 1990 and recorded as receiving radiotherapy the RR, left‐*versus*‐right, was raised by a similar amount for deaths attributed to ischaemic heart disease (RR 1.12, 95% CI 1.08–1.17) and for deaths attributed to all other types of cardiac disease combined (RR 1.12, 95% CI 1.05–1.20, Supporting Information Fig. S6). When specific types of heart disease other than ischaemic heart disease were considered, there were significant increases in women with left‐sided breast cancer compared to right‐sided breast cancer for deaths attributed to arrhythmias (RR 1.24, 95% CI 1.04–1.48), heart failure (RR 1.12, 95% CI 1.01–1.24) and nonrheumatic valvular disease (RR 1.22, 95% CI 1.00–1.48). For ischaemic heart disease, the relative increase in left‐sided breast cancer was larger in women who were younger when their cancer was diagnosed (RR <60 years 1.21, 60+ years 1.08 2*p*_difference_ =0.008), while for all other types of heart disease the relative increases were similar (RR <60 years 1.13, 60+ years 1.11 2*p*_difference_ =0.82; Supporting Information Fig. S7).

Among women diagnosed in 1990 or later and irradiated, the RR left‐*versus*‐right was close to one for ischaemic heart disease and for all other types of cardiac disease and it did not differ significantly from one for any of the individual cardiac diseases considered (Supporting Information Fig. S6). When women whose cancer was diagnosed before age 60 were considered separately the RR left‐*versus*‐right was significantly raised for ischaemic heart disease (RR 1.21, 95% CI 1.00–1.48), while for all other types of heart disease the increase did not reach statistical significance (RR 1.15 95% CI 0.89–1.48; Supporting Information Fig. S7).

## Discussion

In our study of 2 million women treated for breast cancer in 22 countries, there was evidence that radiotherapy, chemotherapy and endocrine therapy were all prescribed selectively, at least among older patients, with lower cardiac mortality among those who received each one of these treatments than among those who did not. This is probably because older women are more likely to have had unfavourable comorbidity profiles at the time of their breast cancer diagnosis, and women who are unfit are less likely to receive treatment. For radiotherapy, there was also evidence of selection according to laterality in women whose breast cancer was diagnosed after 1990, as women with cancer of the left breast were slightly less likely to receive radiotherapy than women with cancer of the right breast. Selective prescribing complicates the interpretation of comparisons of cardiac disease in women who received these treatments with those who did not. However, in women recorded as receiving radiotherapy before 1990, there was no evidence of selection for radiotherapy according to laterality. Therefore, as radiotherapy tends to deliver higher cardiac doses in left breast cancer than in right breast cancer, analyses of cardiac mortality in women with left‐sided *versus* right‐sided cancer diagnosed before 1990 enabled assessment of the causal effect of higher *versus* lower heart dose on cardiac mortality. In these women, cardiac mortality was higher in left‐sided than in right‐sided cancer. The relative increase in risk due to the radiation exposure was larger in women with younger age, it lasted into the third decade after exposure, and was greater in women who also received chemotherapy.

### Patient selection

In observational studies of cancer survivors, comparisons of cardiac mortality in women who did and did not receive various treatments may be dominated by the factors that determine which women are selected for the treatment, rather than the effects of the treatment on the heart.^8^ For radiotherapy, patient selection in our study varied with type of surgery and age (Fig. 1). When women who were recorded as receiving radiotherapy were compared to women who were not recorded as receiving it, radiotherapy after BCS was associated with a substantial deficit in cardiac mortality (RR, irradiated *versus* not: 0.70) whereas radiotherapy after mastectomy was associated with a substantial excess (RR, irradiated *versus* not: 1.24). This pattern was present both overall, and within different geographical regions (Supporting Information Fig. S1). It is not plausible that radiotherapy prevents cardiac disease after BCS and that it causes cardiac disease after mastectomy. Rather, the difference is likely to be due to the fact that, after BCS, guidelines recommend that most women should receive radiotherapy.^38^ Therefore, women who do not receive it are more likely to be unfit, with poor cardiac health, than those who do receive it. In contrast, after mastectomy, most women do not receive radiotherapy, as postmastectomy radiotherapy is recommended only for a minority of women with more advanced cancers.^38^

The effects of patient selection were also seen when women who received systemic therapies were compared to women who did not. Chemotherapy and endocrine use were associated with deficits in cardiac mortality in older women but not in younger women (Fig. 1). This is probably because women with pre‐existing heart disease, or risk factors for heart disease, are less likely to be prescribed chemotherapy or endocrine therapy, and most of these women would have been in the older age group.

In breast cancer radiotherapy, comparison of heart disease rates for left‐sided *versus* right‐sided cancer in women recorded as receiving radiotherapy may reveal the causal effects of higher *versus* lower cardiac radiation doses, but only if laterality did not affect the decision to give radiotherapy, as was the case for women diagnosed before 1990 (Table 2). After 1990, radiotherapy was slightly less often prescribed in left‐sided than in right‐sided cancers, and left‐*versus*‐right differences in cardiac mortality in women diagnosed after 1990 reflect the effect of patient selection and also the consequences of reductions in the typical difference between the cardiac dose in left‐sided and right‐sided breast cancer through the use of more modern radiotherapy techniques by many centres.^39^ In addition, for women diagnosed before 1990, the largest cardiac mortality RRs in women with left‐sided breast cancer compared to right‐sided occurred more than 15 years after diagnosis (Fig. 3). However, few women diagnosed after 1990 had been followed for more than 15 years, and none for more than 25 years. Therefore, data on women diagnosed after 1990 could not be used to infer the causal effects of radiotherapy.

### Interpretation of left‐*versus*‐right cardiac mortality ratios

As a registry‐based study, there are several limitations to the data available, including uncertainty as to whether the therapies recorded were actually administered, and a lack of information on any subsequent therapies received and on the comorbidity profiles of the patients. In addition, only a few of the registries were able to provide information on the use of chemotherapy, which limits the power to study radiotherapy in combination with chemotherapy. However, none of these factors are likely to vary according to the laterality of the breast cancer. Furthermore, available information from other studies suggests that the relative increase in heart disease risks per Gray (Gy) radiation dose to the heart is similar in women with and without heart disease or cardiovascular risk factors prior to radiotherapy.^14^, ^15^, ^40^ So, although prior cardiac disease affects a woman's absolute radiation‐related risk of cardiac mortality, it would not be expected to alter our main results concerning left‐*versus*‐right cardiac mortality RRs.

Three factors need to be considered in the interpretation of left‐*versus*‐right cardiac RRs in women diagnosed with cancer before 1990 and recorded as receiving radiotherapy. First, the left‐*versus*‐right comparison gives information on the risks of higher‐*versus*‐lower radiation dose, not on the risks of radiotherapy *versus* no radiotherapy. This is because irradiation of right breast cancer usually involves some cardiac exposure and hence some cardiac hazard.^39^, ^41^ Therefore, any increase in the risk of heart disease from radiotherapy is likely to be higher than indicated by the left‐*versus*‐right cardiac mortality RRs.

Second, left‐*versus*‐right cardiac mortality RRs in different groups of women recorded as receiving radiotherapy are likely to reflect differences in their left‐minus‐right heart doses which vary according to technique, beam energy and anatomical regions irradiated. For example, in the UK, tangential radiotherapy has been commonly used, for which the left‐minus‐right mean heart dose difference in pre‐2000s radiotherapy was around 3 Gy, while in Denmark electron fields were used during the 1970s and 1980s, for which the left‐minus‐right mean heart dose difference was only around 1–2 Gy.^41^ In contrast, for megavoltage nodal radiotherapy fields used in some countries, the left‐minus‐right dose difference was >5 Gy.^41^ Therefore, variations in left‐*versus*‐right cardiac mortality RRs for particular groups of women may be explained partly by the use of different techniques.

Third, the possibility that left‐minus‐right cardiac dose differences vary with the patient's age within the calendar year and geographical categories used to define the strata in our analyses must be considered. Radiotherapy techniques, beam energies available and regions irradiated may vary from country to country and may change over time. However, within a single country and within a given 5‐year calendar period they do not usually vary according to the patient's age. Direct evidence for this comes from two studies where heart doses in women irradiated for breast cancer at different ages have been reported, and which found no evidence that heart radiation dose varied with age^6^, ^14^ (Supporting Information Table S6). In our study, individual patient regimen information was unavailable so we were unable to assess the dependence of left‐*versus*‐right cardiac mortality RRs on left‐minus‐right heart dose differences directly.

### Comparison with other studies

Cardiac mortality in women irradiated for left‐sided breast cancer has been compared to cardiac mortality in women irradiated for right‐sided breast cancer in over 20 published studies, including around a million women treated since the 1950s.^10^, ^11^, ^12^, ^13^, ^16^, ^19^, ^20^, ^21^, ^22^, ^23^, ^24^, ^25^, ^26^, ^27^, ^28^, ^29^, ^30^, ^31^, ^32^, ^33^, ^34^, ^35^, ^36^, ^37^ Our study is larger than any of these studies, including over 22,000 cardiac deaths in over 1 million irradiated women. In our study, for women recorded as receiving radiotherapy before 1990, the overall cardiac mortality RR, left‐*versus*‐right (1.13) was similar to that in the largest previous study (1.08), based on around 6,000 cardiac deaths in women irradiated for breast cancer in the U.S. Surveillance Epidemiology and End Results (SEER) cancer registries.^19^ No other studies have considered in detail any differences in percent recorded as irradiated for left‐sided and right‐sided cancers, although several have concluded that the cardiac risks have reduced since 1990.

### Cardiac risk according to age irradiated

In our study, there were sufficient cardiac deaths to investigate how the radiation‐related risk varied according to age irradiated. In women recorded as irradiated before 1990, the percent increase in cardiac risk from higher‐*versus*‐lower heart radiation dose was around 50% for women irradiated when aged <40 years, 20% for women irradiated aged 40–59 years and 10% for women irradiated aged 60+ years.

Six previous studies have investigated the cardiac risks of breast cancer radiotherapy according to age irradiated (Supporting Information Table S6). In five of the six, the relative increase in cardiac risk was larger for women irradiated at a younger age, but none of these studies included more than 250 cardiac events in patients irradiated when aged <40 years. In none of the studies was there a statistically significant trend of cardiac risk with age. Three further studies have investigated the effect of age at irradiation in Hodgkin lymphoma where most patients are young adults (Supporting Information Table S7). In one study,^42^ the trend in cardiac RR with age at exposure was statistically significant and in another^40^ the cardiac RR per Gy was greater for younger ages at radiotherapy but numbers were small and the trend was not statistically significant. There are no studies reporting cardiac risk according to age at irradiation in other cancer types.

The highest cardiac disease RRs and risks per Gy for radiation‐related heart disease have been reported in patients irradiated as children or teenagers^43^, ^44^ In a study of cardiac mortality in 4,122 childhood cancer survivors (age at irradiation: 0–14 years) with individual cardiac radiation doses, it was estimated that radiation increased the risk of cardiac death by 60% per Gy (95% CI 20–250),^43^ while in studies of patients irradiated as adults, where the majority of the patients were aged 40–59 years, the increase in heart disease risk per Gy was ~4–7%.^6^, ^14^, ^15^, ^40^ This suggests that the risk per Gy may be around 10 times greater in children than in adults aged 40–59. Our study is the largest to date of radiation‐related cardiac mortality in adult women, including 22,100 cardiac deaths in irradiated women. Analyses of just the women irradiated before 1990 included 14,346 cardiac deaths with 291 in women irradiated aged <40 years and 9,315 in women irradiated at ages 60+. There was, therefore, sufficient power to characterise the effect of higher *versus* lower heart dose according to age at radiotherapy, and the cardiac mortality RRs for women irradiated at ages <40, 40–59 and 60+ were approximately 1.5, 1.2 and 1.1, respectively (Fig. 3 left‐hand panel). Extrapolation from these results suggests that, while the published adult heart radiation dose response relationships of ~4–7% per Gy are likely to apply to the patients irradiated at ages 40–59 years, the risk per Gy for breast cancer patients aged under 40 may be around two and a half times bigger while the risk per Gy for women irradiated at ages 60+ years may be smaller by about a factor of two.

For several other heart disease risk factors, including the effect of smoking *versus* not on myocardial infarction,^45^ and the effect of higher *versus* lower blood pressure and lipids on vascular mortality^46^, ^47^ the relative effect varies with age. Therefore, it is not unexpected to find that the radiation‐related risk also varies with age on a relative scale.

### Cardiac risk from radiotherapy with and without chemotherapy

Our findings suggest that cardiac risks from radiotherapy may be greater in women who also received chemotherapy. Similar findings were reported in a smaller study of 19,464 women irradiated for breast cancer in Denmark in whom the left‐*versus*‐right cardiac incidence RR was 1.32, 95% CI 1.02–1.70 for women who received anthracycline chemotherapy and 1.10, 95% CI 1.01–1.19, in other women.^48^ Anthracycline chemotherapy can itself damage the heart, and these studies suggest it may also potentiate radiation‐related damage.

### Duration of cardiac risk

Several studies have reported that radiation‐related cardiac risk in breast cancer patients lasts for over 20 years^14^, ^19^, ^20^ but, to the best of our knowledge, this is the first to report a significant increase in the period more than 25 years after exposure. Studies of patients irradiated for Hodgkin lymphoma^49^ and childhood cancer^44^, ^50^ have reported radiation‐related cardiac risks lasting for over 30 years. Only further follow‐up of these early cohorts of irradiated breast cancer patients will reveal whether their radiation‐risk also lasts into the fourth decade after exposure.

### Implications for clinical practice

Our study suggests the cardiac risks from breast cancer radiotherapy are greater for younger than for older women, particularly if chemotherapy is also given, and that they last for more than 25 years after treatment. These findings are relevant to young women with node positive breast cancers who are now recommended to receive anthracycline chemotherapy, which can itself damage the heart, and internal mammary node radiotherapy, in which the heart may receive around 8 Gy in left‐radiotherapy and around 4 Gy in right‐radiotherapy.^38^, ^39^ These are comparable to cardiac doses often received prior to the 1990s. For these women, the absolute risks of the radiotherapy need to be balanced against the breast cancer survival benefits.

## Conflict of interest

The authors declare that they have no competing interests.

## Supporting information


**Appendix S1**: Supporting information
