# Supplementary material for: Cardiac mortality after radiotherapy, chemotherapy and endocrine therapy for breast cancer: Cohort study of 2 million women from 57 cancer registries in 22 countries
Source: Int J Cancer. 2020 Mar 4;147(5):1437–49. doi: 10.1002/ijc.32908 (PMC7496256; doi:10.1002/ijc.32908)
Supplement: Supplementary file 1 — Appendix S1: Supporting information [file IJC-147-1437-s001.pdf]

Supplement to: Cardiac Mortality after Radiotherapy, Chemotherapy and Endocrine Therapy for Breast Cancer: Cohort Study of 2 Million Women from 57 Cancer Registries in 22 Countries

KE Henson, P McGale, SC Darby, DM Parkin, Y Wang, CW Taylor (Study conducted in collaboration with the International Association of Cancer Registries)

Contents

Table S1. Registries and other organisations contributing data to the study and individuals who worked on the preparation of the data or who attended the Study Meeting

Text S1. Summary of Data Requested from Individual Cancer Registries

Table S2. Tenth Revision International Classification of Diseases (ICD-10) codes used to define the categories of cardiac disease used in the analysis.

Table S3. Data included in analyses.

Figure S1. Irradiated versus unirradiated women: Cardiac mortality rate ratios by geographic region and type of surgery.

Table S4. Treated versus untreated women: Cardiac mortality rate ratios with and without adjustment for the effect of the other treatments in women aged 60+ years when diagnosed with cancer.

Table S5. Numbers of women with left or right-sided breast cancer, numbers and percentages irradiated by patient and tumour factors.

Figure S2. Irradiated women with left-sided breast cancer versus irradiated women with right-sided breast cancer: Cardiac mortality rate ratios by patient, tumour and treatment characteristics and geographic region.

Figure S3. Irradiated women with left-sided breast cancer versus irradiated women with right-sided breast cancer: Cardiac mortality rate ratios, by country.

Figure S4. Irradiated women with left-sided breast cancer versus irradiated women with right-sided breast cancer: Cardiac mortality rate ratios, by diagnosis period and country.

Figure S5. Figure S5. Irradiated women with left-sided breast cancer versus irradiated women with right-sided breast cancer: Cardiac mortality rate ratios in women who were diagnosed with breast cancer in or after 1990, by age at diagnosis of breast cancer and other factors.

Figure S6. Irradiated women with left-sided breast cancer versus irradiated women with right-sided breast cancer: Cardiac mortality rate ratios, by calendar year of breast cancer diagnosis and certified cause of death.

Figure S7. Irradiated women with left-sided breast cancer versus irradiated women with right-sided breast cancer: Cardiac mortality rate ratios, by age at diagnosis and whether the women were diagnosed with ischaemic heart disease.

Table S6. Breast cancer radiotherapy: Studies reporting cardiac risk according to age irradiated

Table S7. Hodgkin lymphoma radiotherapy: Studies reporting cardiac risk according to age irradiated

Table S1. Registries and other organisations contributing data to the study and individuals who worked on the preparation of the data or who attended the Study Meeting

Country	Organisation	Individuals who worked on the Preparation of the Data or attended the Study Meeting
**Europe: Britain and Ireland**		
UK	*Eastern Cancer Registration & Information Centre (Cambridge)	David Greenberg, Clement Brown, Karen Wright
	*Northern and Yorkshire Cancer Registry and Information Service	David Forman, Phillip Deer, Michael Walkley
	Scottish Cancer Registry	David Brewster, Catherine Storey
	*Thames Cancer Registry	Henrik Møller, Karen Linklater
	*West Midlands Cancer Intelligence Unit	Stacey Croft, Rosie Day
	**Currently part of Public Health England*	
Ireland	Ireland National Cancer Registry	Harry Comber
**Europe: Nordic counties**		
Denmark	Danish Breast Cancer Cooperative Group	Maj-Britt Jensen; Marianne Ewertz
	Danish Cancer Society - Danish Cancer Registry	Georg Paludan Müller, Gerda Engholm, Hans cynthia
Finland	Finnish Cancer Registry	Eero Pukkala
Norway	Cancer Registry of Norway	Steinar Tretli
Sweden	Gothenburg Regional Cancer Registry	Nils Conradi, Ingmarie Johanson
	Regional Tumour Registry, Linköping	Bo Nordenskjöld, Helena Fohlin, Johan Rosell
	The Swedish Cancer Registry (Stockholm & Umea)	Jan Adolfsson, Nils Olof Bengtsson, Per Hall, Anna Bennet
**Europe: Other countries**		
Austria	Cancer Registry of Salzburg	Richard Greil, Johann Lettner
	Cancer Registry of Tyrol	Wilhelm Oberaigner
	Cancer Registry of Voralberg	Hans Concin, Hanno Ulmer

Table S1 continued overleaf

Table S1 continued

Country	Organisation	Individuals who worked on the Preparation of the Data or attended the Study Meeting
Estonia	Estonian Cancer Registry	Margit Mägi, Mare Tekkel, Kaja Rahu
France	Institut Gustave Roussy	Carole Rubino
Germany	Population Based Cancer Registry Bavaria	Gabriele Schubert-Fritschle, Martin Meyer
	Gemeinsames Krebsregister (Berlin)	Bettina Eisinger, Roland Stabenow
	Bremen Cancer Registry	Klaus Giersiepen
	Cancer Registry of Schleswig-Holstein Registerstelle	Alexander Katalinic
Italy	Friuli Venezia Giulia Cancer Registry	Luigino Dal Maso, Lorenzo Simonato, Loris Zanier, Diego Serraino, Margherita de Dottori, Massimo Rugge
	Latina Cancer Registry	Fabio Pannozzo, Antonella Fontana
	Lombardy Cancer Registry	Giovanna Tagliabue, Paolo Contiero, Sabrina Fabiano, Paolo Crosignani
	Modena Cancer Registry	Massimo Federico, Monica Pirani
	National Tumour Institute (Milan)	Russo Antonio
	Parma Province Cancer Registry	Vincenzo De Lisi, Paolo Sgargi
	Piedmont Cancer Registry	Roberto Zanetti, Stefano Rosso
	Ragusa Cancer Registry	Rosario Tumino, Aurora Sigona
	Sondrio Cancer Registry	Roberto Tessandori
	Tuscany Cancer Registry	Lorenzo Livi, Emanuelle Crocetti
	Umbria Cancer Registry	Cynthia Aristei, Francesco La Rosa, Fabrizio Stracci
	Venetian Tumour Registry	Paola Zambon
Lithuania	Lithuanian Cancer Registry	Juozas Kurtinaitis, Simona Letautiene
Netherlands	Eindhoven Cancer Registry	Isabelle Soerjomataram
	Netherlands Cancer Institute	Flora van Leeuwen, Berthe Aleman, Willem Klokman
Slovakia	National Cancer Registry of Slovak Republic	Martina Ondrusova

Table S1 continued overleaf

Table S1 continued

Country	Organisation	Individuals who worked on the Preparation of the Data or attended the Study Meeting
Slovenia	Cancer Registry of Slovenia	Maja Primic Zakelj
Spain	Catalan Institute of Oncology (Barcelona)	Josepa Ribes Puig
	Epidemiology Unit and Girona Cancer Registry	Rafael Marcos-Gragera
	Navarra Cancer Registry	Maria Eva Ardanaz Aicua
Switzerland	Geneva Cancer Registry	Christine Bouchardy, Massimo Usel
	Grisons Cancer Registry	Bertrand Camey, Harald Frick
	Cancer Registry of St. Gall-Appenzell	Silvia Ess
	Ticino Cancer Registry	Andrea Bordoni, Alessandra Spitale
**North America**		
Canada	Alberta Cancer Registry	Heather Bryant
	British Columbia Cancer Registry	Ivo Olivotto, Caroline Speers
	Ontario Cancer Registry	Laurence Paszat
USA	Connecticut Tumour Registry	Anthony Polednak
	Puerto Rico Central Cancer Registry	Mariela Torres Cintron
	Surveillance Epidemiology and End Results Program	Brenda Edwards
**Other regions**		
Australia	New South Wales Central Cancer Registry	Paul Jelfs, Elizabeth Tracey, Helen Barraclough
Israel	Israel National Cancer Registry	Micha Barchana
Japan	Tumour & Tissue Registry Office (Hiroshima)	Nobue Nishi, Ritsu Sakata
	Kanagawa Cancer Centre	Naoyuke Okamoto
	Nagasaki Prefectural Cancer Registry	Midori Soda

Note: Several other organisations provided preliminary data for the study, but did not have information on all the variables necessary for inclusion in the final analysis.

Text S1. Summary of Data Requested from Individual Cancer Registries

Cancer registries and other appropriate organisations in high income countries worldwide were invited to contribute to the study. Appropriate contacts were identified through the International Association of Cancer Registries and personal knowledge. A Data Sheet was made available to each organisation detailing the eligibility and exclusion criteria and the variables requested (see next page). Each contributing organisation assembled a patient-level data file locally including follow-up information. The file was then depersonalised before being transferred to Oxford, alongside the coding sheet used for any local variables. The data files were then collated and combined by the analysis team in Oxford.

Eligibility criteria:

1. Women diagnosed with carcinoma in situ in the breast, who received radiotherapy.
2. Women diagnosed with invasive breast cancer (regardless of whether they received radiotherapy) and who were not already in 1.

The term "index breast cancer" denoted the first event (either carcinoma in situ or invasive cancer) after the start of the registry that qualified the woman for entry to the study.

Exclusion criteria:

– Male sex
– Bilateral breast cancer or carcinoma in situ
– Previous invasive cancer (apart from non-melanoma skin cancer) on or before the date of diagnosis of the index breast cancer. A previous diagnosis of cancer in situ of a site other than the breast (e.g. carcinoma in situ of cervix uteri) was allowed
– Emigration at any time prior to date of diagnosis of the index breast cancer (even if subsequently re-immigrated)
– Immigration at any time (before or after date of index breast cancer diagnosis)
– Death on recorded date of diagnosis of the index breast cancer, or cancer diagnosed at autopsy
– Previous treatment with thoracic radiotherapy

Text S1 (continued)

Variable	Values recorded
**Essential variables**	
ID number	(depersonalised)
Date of birth (or age at diagnosis of index breast cancer)	DD/MM/YYYY
Date of diagnosis of index breast cancer	DD/MM/YYYY
Laterality of index breast cancer	Left / Right / Unknown / Bilateral*
Behaviour of index breast cancer	Malignant / In situ
Radiotherapy for index breast cancer†	Yes / No / Unknown
Date of death or emigration or last known to be alive, or date lost to follow-up	DD/MM/YYYY or Unknown
Status on date above	Dead / Emigrated / Alive / Unknown
Underlying cause of death	Code ‡
**Desirable variables**	
Region/hospital	Country specific
Ethnic group	Codes used by cancer registry
Topography of breast cancer	Code ¶
Morphology of breast cancer	Code ¶
ER-status	Codes used by cancer registry
PR-status	Codes used by cancer registry
Stage of breast cancer	CIS § / Local ** / Regional †† / Advanced ‡‡ / Unknown
Type of surgery to breast	Breast Conserving Surgery / Mastectomy / Other / None / Unknown
Type of surgery to the axilla	Axillary node clearance / Axillary node sampling / Other / None / Unknown
Chemotherapy	Yes / No / Unknown
Endocrine therapy	Yes / No / Unknown
Ovarian ablation or suppression	Yes / No / Unknown
Dates of diagnosis of any subsequent invasive cancers (including invasive breast cancer, if recorded)	DD/MM/YYYY
Site of any subsequent invasive cancers	Code ‡
Laterality of any subsequent invasive lung or breast cancer	Left / Right / Unknown / Not applicable ¶ ¶ / Bilateral *
Morphology of any subsequent invasive cancer	Code ¶
Basis of diagnosis of any subsequent invasive cancer	Codes used by cancer registry
Contributory causes of death (all known)	Code(s) ‡

* Diagnosed in both breasts simultaneously (or within 4 months)
† If possible, distinguishing between women who received radiotherapy and those for whom it was indicated but not necessarily received
‡ Coding schedule requested (for example ICD 7/8/9/10 or registry-specific code)
¶ Coding schedule requested (for example ICD-0 code, edition 1, 2, or 3, or registry-specific code)
§ Carcinoma in situ
** Node-negative
†† Node-positive or locally advanced
‡‡ Distant metastases
¶ ¶ If diagnosed in the trachea

Table S2. Tenth Revision International Classification of Diseases (ICD-10) codes used to define the categories of cardiac disease used in the analysis.

Cause of Death	ICD-10 codes
Ischaemic heart disease	I20-I25
Arrhythmia	I44-49
Heart failure	I01.2, I09.0, I11.0, I13.0, I13.2, I40-I43, I50, I51.4-5, I51.7
Non-rheumatic valvular	I34-I39
Rheumatic valvular	I01.1, I05-I08, I09.1
Pericarditis	I01.0, I09.2, I30-I32
Other cardiac disease	I01.8-9, I02.0, 109.8-9, I11.9, I13.1, I13.9, I27.1-9, I33, I51.0-3, I51.6, I51.8-9, I52
**All cardiac disease**	**I01, I02.0, I05-I09, I11, I13, I20-I25, I27.1-9, I30-I52**

Table S3. Data included in analyses.

	Organisation	Dates of breast cancer diagnosis	Period of follow-up	Number of women	Number given RT	% RT	Cardiac disease deaths
**Europe: Britain and Ireland**							
UK	Eastern Cancer Registration & Information Centre (Cambridge)	1941 to 2006	1969 to 2007	43,544	23,266	53	628
	Northern and Yorkshire Cancer Registry and Information Service	1980 to 2004	1991 to 2006	51,701	29,723	57	597
	Scottish Cancer Registry	1996 to 2004	1997 to 2006	22,486	13,373	59	389
	Thames Cancer Registry	1960 to 2006	1960 to 2006	160,472	81,370	51	2,642
	West Midlands Cancer Intelligence Unit	1977 to 2004	1977 to 2007	65,781	39,470	60	1,929
Ireland	Ireland National Cancer Registry	1994 to 2004	1994 to 2006	13,594	6,117	45	114
**Europe: Nordic counties**							
Denmark	Danish Breast Cancer Cooperative Group	1977 to 2005	1978 to 2011	57,663	28,529	49	2,046
	Danish Cancer Registry	1943 to 1977	1943 to 2001	44,217	30,923	70	3,632
Finland	Finnish Cancer Registry	1953 to 2006	1969 to 2006	76,194	45,850	60	5,310
Norway	Cancer Registry of Norway	1986 to 2004	1986 to 2006	20,310	9,644	47	187
Sweden	Gothenburg Regional Cancer Registry	1989 to 2006	1989 to 2007	12,651	6,925	55	279
	Regional Tumour Registry, Linköping	1986 to 2003	1986 to 2004	7,077	5,209	74	212
	The Swedish Cancer Registry (Stockholm & Umea)	1976 to 2006	1976 to 2006	28,332	16,414	58	1,262

Table S3 continued overleaf

Table S3 continued

	Organisation	Dates of breast cancer diagnosis	Period of follow-up	Number of women	Number given RT	% RT	Cardiac disease deaths
**Europe: Other countries**							
Austria	Cancer Registry of Salzburg	1962 to 2007	1979 to 2007	6,011	3,556	59	91
	Cancer Registry of Tyrol	1988 to 2003	1988 to 2005	4,138	1,812	44	114
	Cancer Registry of Voralberg	1985 to 2003	1985 to 2007	1,459	1,320	90	18
Estonia	Estonian Cancer Registry	1968 to 2000	1968 to 2005	11,043	4,959	45	722
France	Institut Gustave Roussy	1954 to 1984	1954 to 2005	6,017	4,472	74	192
Germany	Population Based Cancer Registry Bavaria	1998 to 2007	1998 to 2007	30,954	23,545	76	55
	Gemeinsames Krebsregister (Berlin)	1961 to 2005	1961 to 2007	194,532	101,509	52	2,170
	Bremen Cancer Registry	1988 to 2005	1999 to 2007	1,935	1,884	97	5
	Cancer Registry of Schleswig-Holstein Registerstelle	1997 to 2004	1999 to 2007	9,425	7,508	80	53
Italy	Friuli Venezia Giulia Cancer Registry	1995 to 2003	1995 to 2005	516	516	100*	2
	Latina Cancer Registry	2002 to 2007	2004 to 2007	489	487	100*	0
	Lombardy Cancer Registry	1980 to 2000	1980 to 2005	3,015	533	18	38
	Modena Cancer Registry	1988 to 2005	1989 to 2007	5,074	2,494	49	48
	National Tumour Institute (Milan)	1999 to 2002	1999 to 2006	4,306	2,924	68	44
	Parma Province Cancer Registry	1994 to 2004	1994 to 2006	1,993	1,767	89	19
	Piedmont Cancer Registry	2000 to 2003	2000 to 2007	2,701	1,828	68	6
	Ragusa Cancer Registry	1990 to 2003	1990 to 2006	830	542	65	15
	Sondrio Cancer Registry	1997 to 2006	1998 to 2007	994	565	57	7
	Tuscany Cancer Registry	1985 to 2004	1985 to 2005	13,758	5,761	42	291
	Umbria Cancer Registry	1968 to 2004	1994 to 2006	3,721	2,040	55	52
	Venetian Tumour Registry	1987 to 2001	1987 to 2007	2,307	1,485	64	33

Table S3 continued overleaf

Table S3 continued

	Organisation	Dates of breast cancer diagnosis	Period of follow-up	Number of women	Number given RT	% RT	Cardiac disease deaths
Lithuania	Lithuanian Cancer Registry	1993 to 2005	1993 to 2007	10,812	4,765	44	192
Netherlands	Eindhoven Cancer Registry	1964 to 2004	1971 to 2004	18,319	11,840	65	0
	Netherlands Cancer Institute	1970 to 1987	1971 to 2004	7,101	6,217	88	235
Slovakia	National Cancer Registry of Slovak Republic	1978 to 2003	1978 to 2006	28,139	18,314	65	73
Slovenia	Cancer Registry of Slovenia	1971 to 2004	1988 to 2007	8,863	3,754	42	135
Spain	Institute Catalá d'Oncolgía (Barcelona)	1991 to 1997	1991 to 2005	850	646	76	15
	Cancer Registry of Girona	1963 to 2005	1978 to 2006	1,818	1,722	95	18
	Navarra Cancer Registry	1990 to 2002	1990 to 2007	3,187	2,332	73	24
Switzerland	Geneva Cancer Registry	1970 to 2004	1975 to 2005	802	630	79	15
	Grisons Cancer Registry	1989 to 2005	1989 to 2007	1,524	591	39	30
	Cancer Registry of St. Gall-Appenzell	1940 to 2005	1980 to 2007	2,689	1,076	40	53
	Ticino Cancer Registry	1995 to 2004	1996 to 2007	1,961	1,178	60	7
**North America**							
Canada	Alberta Cancer Registry	1989 to 2004	1989 to 2007	20,911	10,533	50	411
	British Columbia Cancer Registry	1985 to 2004	1985 to 2007	29,723	19,115	64	606
	Ontario Cancer Registry	1982 to 1997	1984 to 2007	55,477	55,477	100*	1,294
USA	Connecticut Tumour Registry	1935 to 1972	1935 to 2007	26,640	7,852	29	3,379
	Puerto Rico Central Cancer Registry	1987 to 2003	1987 to 2008	13,263	6,982	53	137
	Surveillance Epidemiology and End Results Program	1973 to 2008	1973 to 2008	738,145	327,521	44	24,413

Table S3 continued overleaf

Table S3 continued

	Organisation	Dates of breast cancer diagnosis	Period of follow-up	Number of women	Number given RT	% RT	Cardiac disease deaths
**Other regions**							
Australia	New South Wales Central Cancer Registry	1972 to 2004	1972 to 2004	46,993	27,023	58	845
Israel	Israel National Cancer Registry	1960 to 2000	1961 to 2006	4,827	1,503	31	108
Japan	Tumour & Tissue Registry Office (Hiroshima)	1957 to 2001	1957 to 2007	695	104	15	48
	Kanagawa Cancer Centre	1941 to 1989	1971 to 2007	1,631	372	23	19
	Nagasaki Prefectural Cancer Registry	1956 to 2005	1961 to 2005	638	638	100*	5

*Registry provided information only on women who were given radiotherapy.

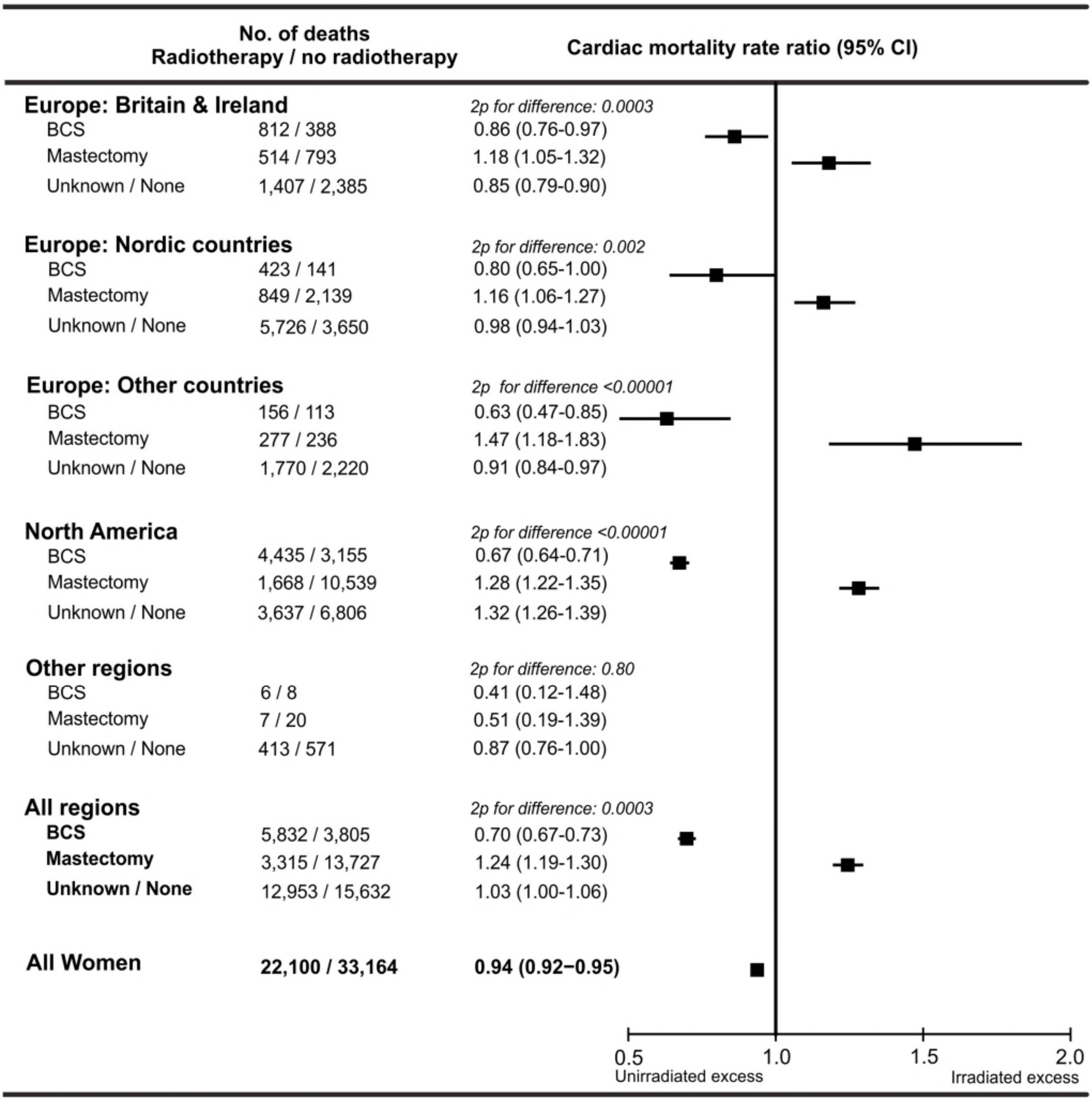

BCS: Breast-conserving surgery
2p is for difference between BCS and mastectomy

Figure S1. Irradiated versus unirradiated women: Cardiac mortality rate ratios by geographic region and type of surgery.

Rate ratios estimated by Poisson regression with stratification by time since breast cancer diagnosis, age at breast cancer diagnosis, calendar year of breast cancer diagnosis, and country.

Table S4. Treated versus untreated women: Cardiac mortality rate ratios with and without adjustment for the effect of the other treatments in women aged 60+ years when diagnosed with cancer.

Treatment	Cardiac mortality rate ratio, treatment versus no treatment (95% CI)	
	**Unadjusted**	**Adjusted for other treatments**
Radiotherapy	0.87 (0.85-0.89)	0.88 (0.86-0.90)
Chemotherapy	0.86 (0.80-0.92)	0.87 (0.81-0.94)
Endocrine therapy	0.92 (0.88-0.95)	0.93 (0.89-0.97)

Rate ratios estimated by Poisson regression with stratification by time since breast cancer diagnosis, age at breast cancer diagnosis, calendar year of breast cancer diagnosis, and country.

Table S5. Numbers of women with left-sided and right-sided breast cancer, numbers of women and percentages irradiated by patient and tumour factors.

		All women						Women diagnosed <1990						Women diagnosed 1990+					
		Number		Percentage irradiated				Number		Percentage irradiated				Number		Percentage irradiated			
		Left	Right	Left	Right	Diff*	p value	Left	Right	Left	Right	Diff*	p value	Left	Right	Left	Right	Diff*	p value
Calendar year of diagnosis	<1970	48,041	44,621	56.16	55.96	0.21	0.53	48,041	44,621	56.16	55.96	0.21	0.53	-	-	-	-	-	-
	1970-1979	86,778	80,753	50.19	50.21	-0.01	0.95	86,778	80,753	50.19	50.21	-0.01	0.95	-	-	-	-	-	-
	1980-1989	176,543	164,497	46.71	46.77	-0.06	0.72	176,543	164,497	46.71	46.77	-0.06	0.72	-	-	-	-	-	-
	1990-1999	317,616	298,183	52.96	53.08	-0.11	0.37							317,616	298,183	52.96	53.08	-0.11	0.37
	2000+	367,307	349,909	55.09	55.50	-0.42	<0.001	-	-	-	-	-	-	367,307	349,909	55.09	55.50	-0.42	<0.001
Age at cancer diagnosis (years)	<40	67,579	66,078	55.14	55.35	-0.21	0.44	24,108	23,421	53.38	53.64	-0.26	0.56	43,471	42,657	56.12	56.30	-0.17	0.61
	40-49	204,149	195,363	55.59	55.50	0.09	0.57	62,804	59,169	53.38	52.86	0.52	0.07	141,345	136,194	56.57	56.64	-0.07	0.70
	50-59	267,959	252,010	56.03	56.25	-0.22	0.11	77,296	71,682	52.62	52.72	-0.10	0.71	190,663	180,328	57.41	57.65	-0.24	0.14
	60-69	261,607	244,036	53.09	53.32	-0.22	0.12	85,023	78,650	49.06	48.89	0.17	0.48	176,584	165,386	55.04	55.42	-0.38	0.02
	70-79	194,991	180,476	42.97	43.26	-0.29	0.07	62,131	56,949	38.98	39.28	-0.30	0.29	132,860	123,527	44.83	45.10	-0.27	0.18
Race/ethnicity																			
	White	469,630	446,365	47.63	47.90	-0.27	0.01	132,602	123,935	38.23	37.89	0.34	0.07	337,028	322,430	51.33	51.75	-0.42	<0.001
	Black	34,502	32,348	42.99	42.89	0.10	0.80	6,150	5,653	29.15	30.23	-1.08	0.20	28,352	26,695	45.99	45.57	0.42	0.32
	Asian/pacific	29,906	28,628	45.27	45.91	-0.64	0.12	3,659	3,514	25.61	26.27	-0.66	0.52	26,247	25,114	48.01	48.65	-0.64	0.14
	Other/unknown	141,446	131,814	52.12	52.29	-0.17	0.38	57,666	53,451	52.06	52.13	-0.06	0.83	83,780	78,363	52.16	52.39	-0.24	0.34
	Not recorded	320,801	298,808	61.66	61.98	-0.32	0.01	111,285	103,318	62.50	62.91	-0.41	0.05	209,516	195,490	61.21	61.48	-0.27	0.08
Stage at cancer diagnosis	DCIS	67,950	63,824	38.12	38.26	-0.14	0.60	5,876	5,420	13.56	14.70	-1.14	0.08	62,074	58,404	40.45	40.45	0.00	0.99
	Local	378,266	360,074	50.04	50.41	-0.37	0.002	88,521	83,116	34.03	33.87	0.15	0.50	289,745	276,958	54.93	55.37	-0.44	<0.001
	Regional	244,231	229,963	54.55	54.65	-0.10	0.51	67,504	63,057	50.40	50.12	0.28	0.32	176,727	166,906	56.14	56.36	-0.22	0.20
	Metastatic/unknown	201,433	186,919	50.27	50.39	-0.13	0.43	96,655	89,279	54.15	54.43	-0.28	0.23	104,778	97,640	46.68	46.70	-0.02	0.93
	Not recorded	104,405	97,183	70.77	71.14	-0.36	0.07	52,806	48,999	67.66	67.97	-0.31	0.29	51,599	48,184	73.96	74.36	-0.40	0.15
Surgery																			
	BCS	262,831	252,280	72.85	72.95	-0.11	0.38	27,879	26,435	70.95	70.13	0.82	0.04	234,952	225,845	73.07	73.28	-0.21	0.10
	Mastectomy	249,487	236,322	26.87	27.05	-0.18	0.15	79,239	74,443	32.44	32.46	-0.02	0.92	170,248	161,879	24.28	24.57	-0.29	0.05
	None/unknown	399,406	370,468	51.13	51.28	-0.15	0.19	162,605	150,201	47.18	47.24	-0.07	0.70	236,801	220,267	53.84	54.03	-0.19	0.21
	Not recorded	84,561	78,893	71.97	72.22	-0.25	0.26	41,639	38,792	74.00	74.21	-0.21	0.50	42,922	40,101	70.01	70.30	-0.29	0.35
Radiotherapy																			
	Yes	523,570	494,935	100.00	100.00	0.00	-	153,004	142,452	100.00	100.00	0.00	-	370,566	352,483	100.00	100.00	0.00	-
	No	472,715	443,028	0.00	0.00	0.00	-	158,358	147,419	0.00	0.00	0.00	-	314,357	295,609	0.00	0.00	0.00	-
Chemotherapy																			
	Yes	115,195	107,882	63.84	63.87	-0.02	0.91	16,717	15,800	58.41	59.09	-0.68	0.21	98,478	92,082	64.77	64.69	0.08	0.72
	No	355,681	329,821	52.72	53.09	-0.37	0.002	150,382	138,696	55.09	55.42	-0.33	0.08	205,299	191,125	50.97	51.40	-0.42	0.01
	Unknown	16,193	15,260	72.16	71.97	0.19	0.70	3,504	3,204	63.98	62.64	1.34	0.25	12,689	12,056	74.42	74.44	-0.03	0.96
	Not recorded	509,216	485,000	49.26	49.47	-0.21	0.03	140,759	132,171	41.31	41.04	0.27	0.15	368,457	352,829	52.30	52.63	-0.34	0.004
Endocrine therapy																			
	Yes	164,624	152,995	62.80	63.15	-0.36	0.04	31,797	29,199	60.86	61.26	-0.40	0.31	132,827	123,796	63.26	63.60	-0.34	0.08
	No	289,196	268,949	50.73	51.07	-0.35	0.01	136,210	126,040	54.05	54.37	-0.33	0.09	152,986	142,909	47.77	48.16	-0.39	0.03
	Unknown	8,175	7,307	69.50	69.39	0.12	0.87	2,147	1,953	58.64	56.53	2.11	0.17	6,028	5,354	73.37	74.08	-0.70	0.40
	Not recorded	534,290	508,712	50.12	50.30	-0.18	0.07	141,208	132,679	41.62	41.40	0.23	0.23	393,082	376,033	53.18	53.44	-0.26	0.02
**Total**		**996,285**	**937,963**	**52.55**	**52.77**	**-0.21**	**0.003**	**311,362**	**289,871**	**49.14**	**49.14**	**<0.01**	**0.98**	**684,923**	**648,092**	**54.10**	**54.39**	**-0.28**	**0.001**

* Difference between percentage irradiated left and right

	Number of deaths Left-sided / right-sided	Cardiac mortality rate ratio (95% CI)
**Calendar year of diagnosis**		*2p for trend <0.0001*
<1970	1,864 / 1,573	1.11 (1.04−1.19)
1970−79	2,629 / 2,111	1.18 (1.11−1.25)
1980−89	3,319 / 2,850	1.10 (1.05−1.16)
1990−99	2,945 / 2,857	0.96 (0.91−1.01)
2000+	1,006 / 946	1.02 (0.93−1.12)
**Age at cancer diagnosis**		*2p for trend <0.0001*
<40	201 / 139	1.39 (1.11−1.73)
40−49	1,012 / 850	1.15 (1.05−1.26)
50−59	2,265 / 1,775	1.20 (1.13−1.28)
60−69	4,091 / 3,668	1.05 (1.00−1.10)
70−79	4,194 / 3,905	1.01 (0.97−1.05)
**Time since diagnosis (years)**		*2p for trend <0.0001*
0−4	3,370 / 3,144	1.00 (0.96−1.05)
5−14	5,141 / 4,582	1.05 (1.01−1.10)
15−24	2,219 / 1,784	1.18 (1.11−1.26)
25+	1,033 / 827	1.21 (1.10−1.33)
**Race/Ethnicity**		*2p for heterogeneity: 0.74*
White	6,036 / 5,376	1.06 (1.03−1.10)
Black	435 / 371	1.12 (0.98−1.29)
Asian/Pacific	165 / 152	1.10 (0.88−1.38)
Other/unknown	1,176 / 949	1.13 (1.04−1.23)
Not recorded	3,951 / 3,489	1.06 (1.01−1.11)
**Stage**		*2p for heterogeneity: 0.07*
DCIS	245 / 195	1.16 (0.96−1.41)
Local	4,202 / 3,764	1.05 (1.01−1.10)
Regional	3,118 / 2,684	1.12 (1.06−1.18)
Metastatic/unknown	1,650 / 1,475	1.05 (0.98−1.13)
Not recorded	2,548 / 2,219	1.06 (1.00−1.12)
**Surgery**		*2p for difference: 0.14*
BCS	2,998 / 2,834	1.01 (0.96−1.06)
Mastectomy	1,766 / 1,549	1.07 (1.00−1.15)
None/unknown	4,619 / 3,845	1.13 (1.08−1.18)
Not recorded	2,380 / 2,109	1.06 (1.00−1.12)
**Chemotherapy**		*2p for difference: 0.25*
Yes	413 / 352	1.17 (1.00−1.35)
No	4,475 / 3,951	1.06 (1.02−1.11)
Unknown	113 / 87	1.18 (0.89−1.58)
Not recorded	6,762 / 5,947	1.07 (1.03−1.11)
**Endocrine therapy**		*2p for difference: 0.14*
Yes	1,384 / 1,266	1.02 (0.94−1.10)
No	3,473 / 3,020	1.09 (1.04−1.14)
Unknown	73 / 51	1.23 (0.85−1.78)
Not recorded	6,833 / 6,000	1.07 (1.04−1.11)
**Region**		*2p for heterogeneity: 0.86*
Europe: Britain & Ireland	1,494 / 1,239	1.11 (1.03−1.20)
Europe: Nordic countries	3,711 / 3,287	1.06 (1.01−1.11)
Europe: Other countries	1,170 / 1,033	1.08 (0.99−1.18)
North America	5,155 / 4,585	1.06 (1.02−1.11)
Other regions	233 / 193	1.06 (0.87−1.29)
**All women**	**11,763 / 10,337**	**1.07 (1.04−1.10)**

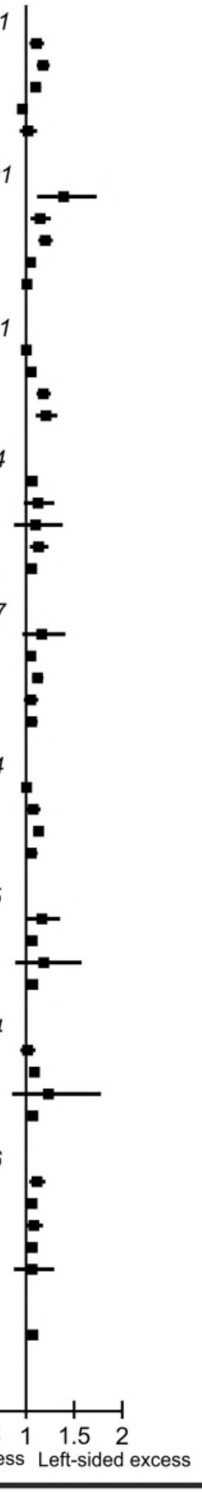

DCIS: Ductal carcinoma in situ.
BCS: Breast-conserving surgery.
p-values are for tests of trend or heterogeneity between categories with known values
(i.e. excluding Other/unknown, Not recorded, Metastatic/unknown, None/unknown)

Figure S2. Irradiated women with left-sided breast cancer versus irradiated women with right-sided breast cancer: Cardiac mortality rate ratios by patient, tumour and treatment characteristics, and geographic region.

Rate ratios estimated by Poisson regression with stratification by time since breast cancer diagnosis, age at breast cancer diagnosis, calendar year of breast cancer diagnosis, and country. See Fig. 3 in the main text for separate analyses of women irradiated <1990 and 1990+. Separate results for individual countries are given in Fig. S3.

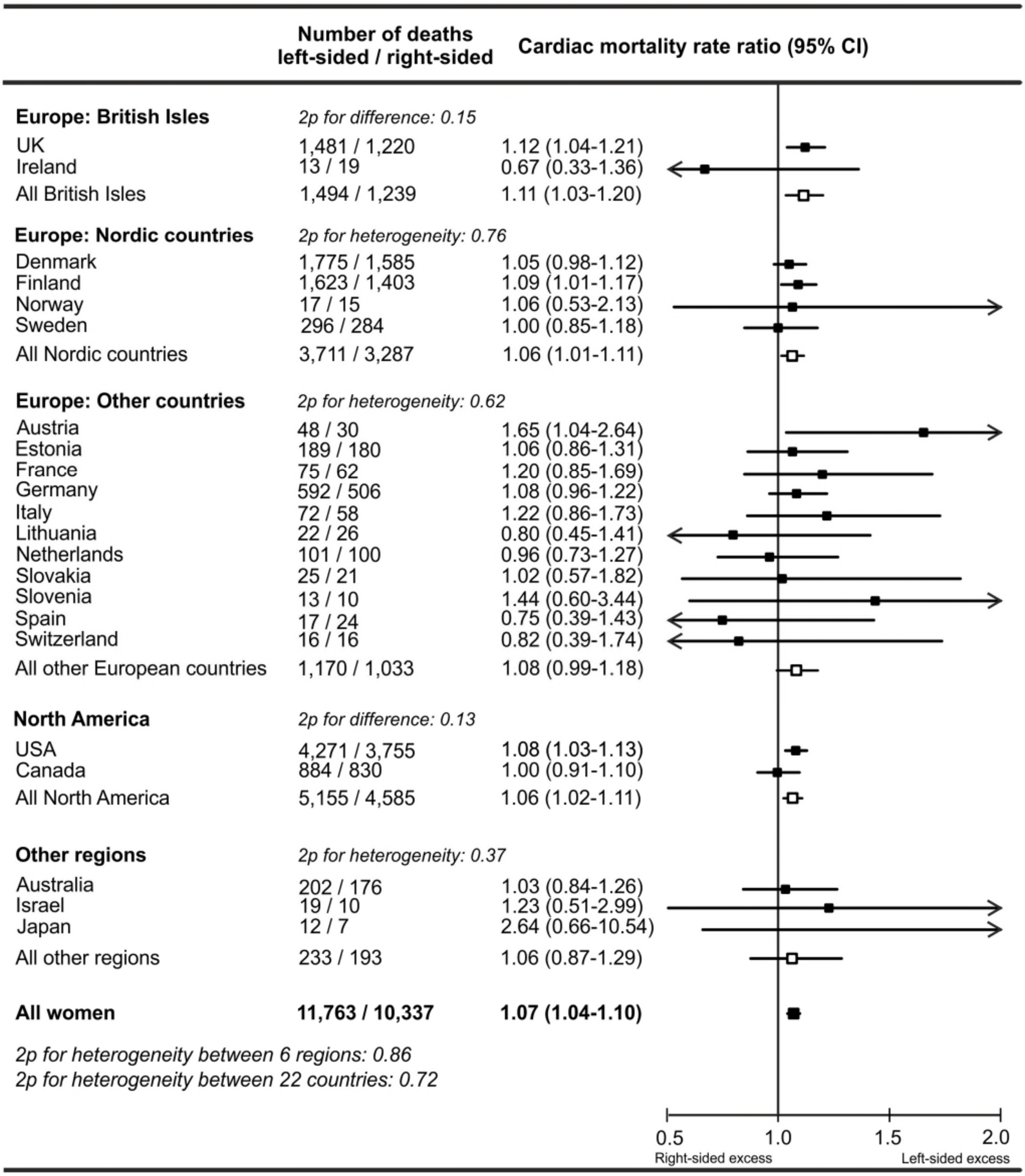

Figure S3. Irradiated women with left-sided breast cancer versus irradiated women with right-sided breast cancer: Cardiac mortality rate ratios, by country.

Rate ratios estimated by Poisson regression with stratification by time since breast cancer diagnosis, age at breast cancer diagnosis, calendar year of breast cancer diagnosis, and country. See Figure S4 for separate analyses for women diagnosed before 1990 and diagnosed 1990+

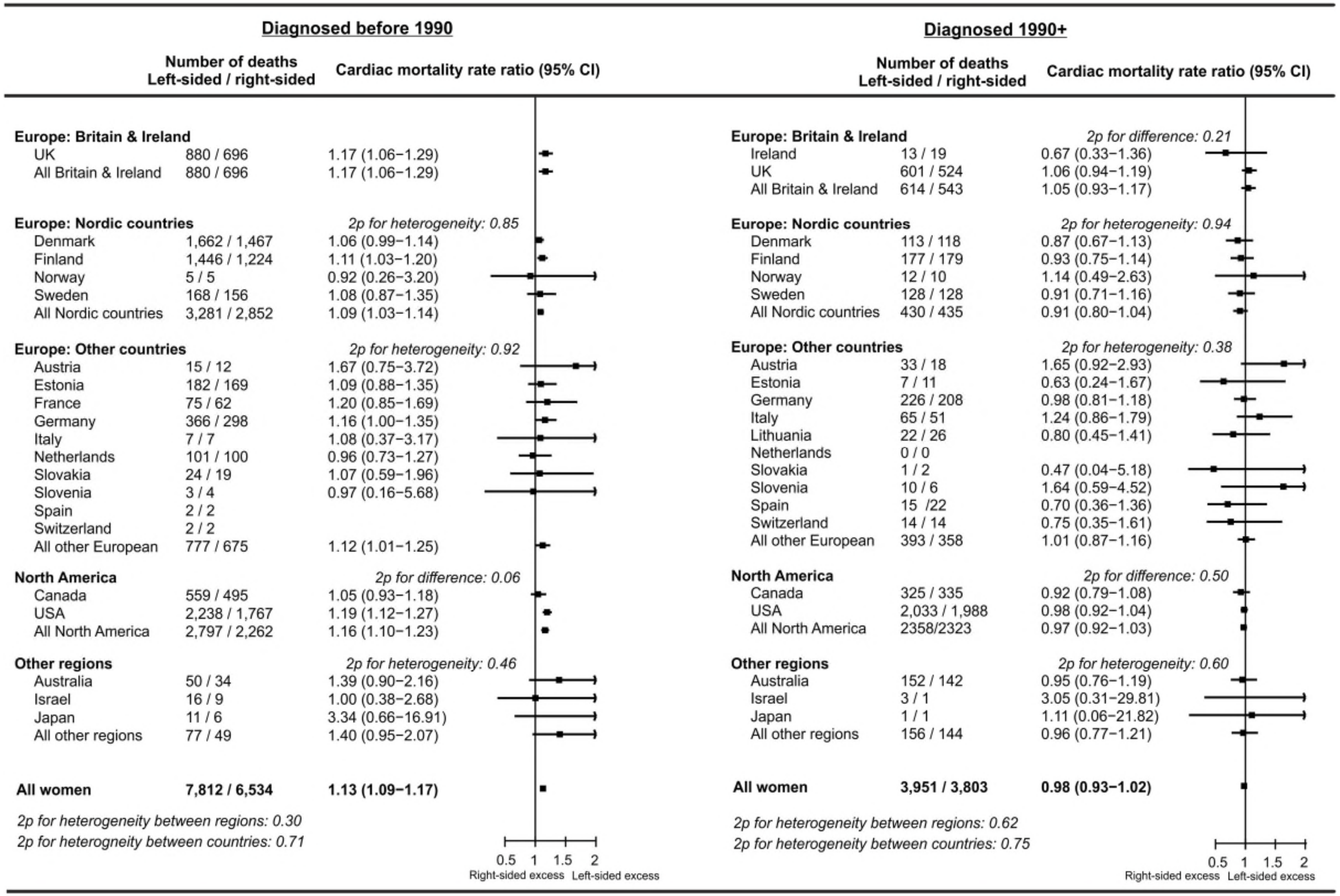

Figure S4. Irradiated women with left-sided breast cancer versus irradiated women with right-sided breast cancer: Cardiac mortality rate ratios, by diagnosis period and country.

Rate ratios estimated by Poisson regression with stratification by time since breast cancer diagnosis, age at breast cancer diagnosis, calendar year of breast cancer diagnosis, and country.

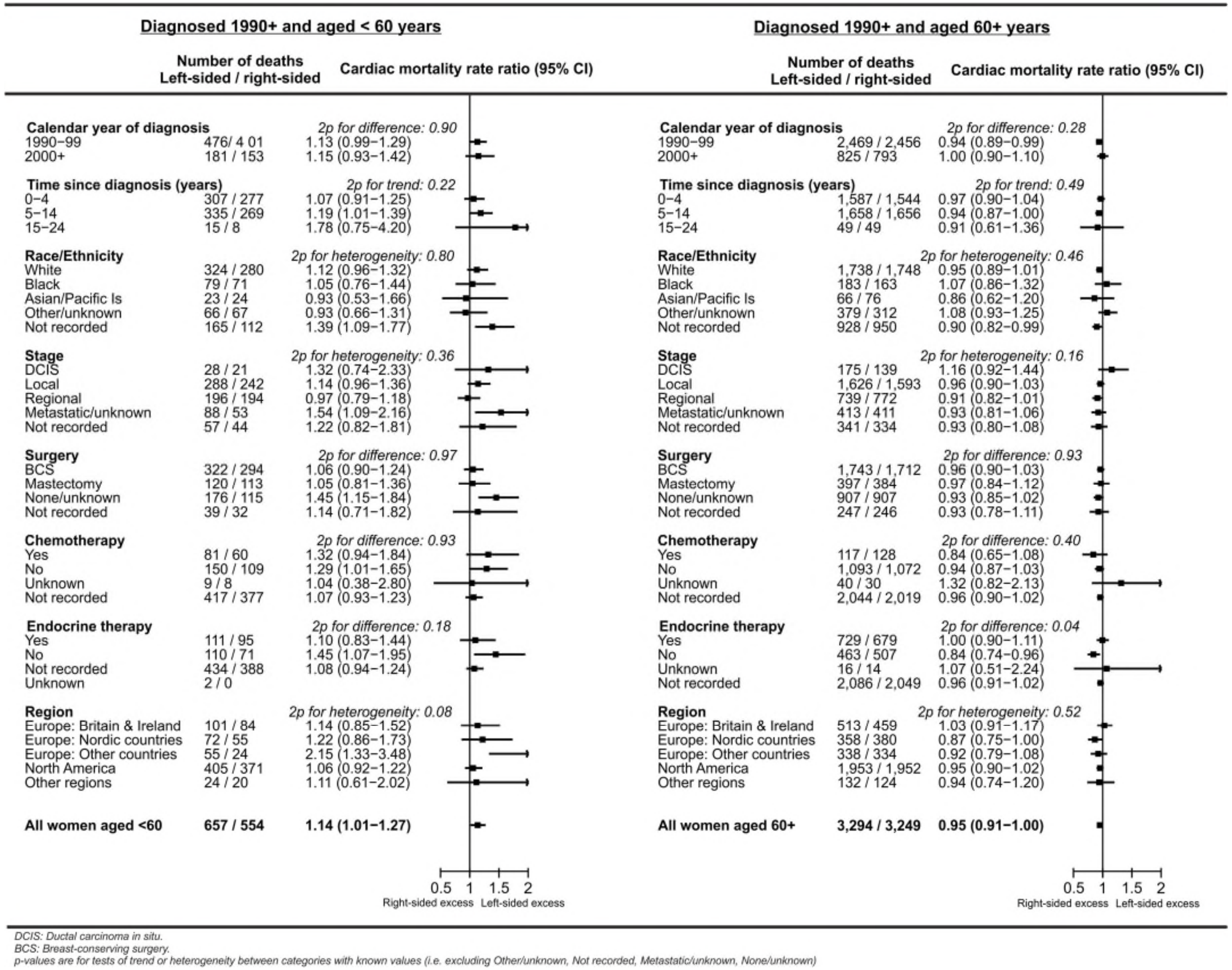

Figure S5. Irradiated women with left-sided breast cancer versus irradiated women with right-sided breast cancer: Cardiac mortality rate ratios in women who were diagnosed with breast cancer in or after 1990, by age at diagnosis of breast cancer and other factors. Rate ratios estimated by Poisson regression with stratification by time since breast cancer diagnosis, age at breast cancer diagnosis, calendar year of breast cancer diagnosis, and country.
[p value for difference in RR for all women aged <60 vs 60+: 0.005]

	Number of deaths Left-sided / right-sided	Cardiac mortality rate ratio (95% CI)
**Diagnosed before 1990**		
Ischaemic heart disease	5,441 / 4,558	1.12 (1.08−1.17)
Other types of cardiac disease	1,908 / 1,617	1.12 (1.05−1.20)
Arrhythmia	291 / 222	1.24 (1.04−1.48)
Heart failure	760 / 644	1.12 (1.01−1.24)
Non−rheumatic valvular disease	233 / 182	1.22 (1.00−1.48)
Rheumatic valvular disease	87 / 73	1.13 (0.83−1.55)
Pericardial disease	19 / 13	1.33 (0.65−2.71)
Other cardiac disease	433 / 388	1.07 (0.93−1.23)
**All cardiac disease**	**7,812 / 6,534**	**1.13 (1.09−1.17)**
**Diagnosed 1990+**		
Ischaemic heart disease	1,640 / 1,536	0.99 (0.92−1.06)
Other types of cardiac disease	758 / 763	0.92 (0.84−1.02)
Arrhythmia	152 / 162	0.88 (0.70−1.09)
Heart failure	331 / 327	0.94 (0.81−1.10)
Non−rheumatic valvular disease	78 / 66	1.10 (0.79−1.53)
Rheumatic valvular disease	26 / 33	0.72 (0.43−1.21)
Pericardial disease	6 / 6	0.88 (0.28−2.74)
Other cardiac disease	116 / 125	0.87 (0.68−1.13)
**All cardiac disease**	**3,951 / 3,803**	**0.98 (0.93−1.02)**

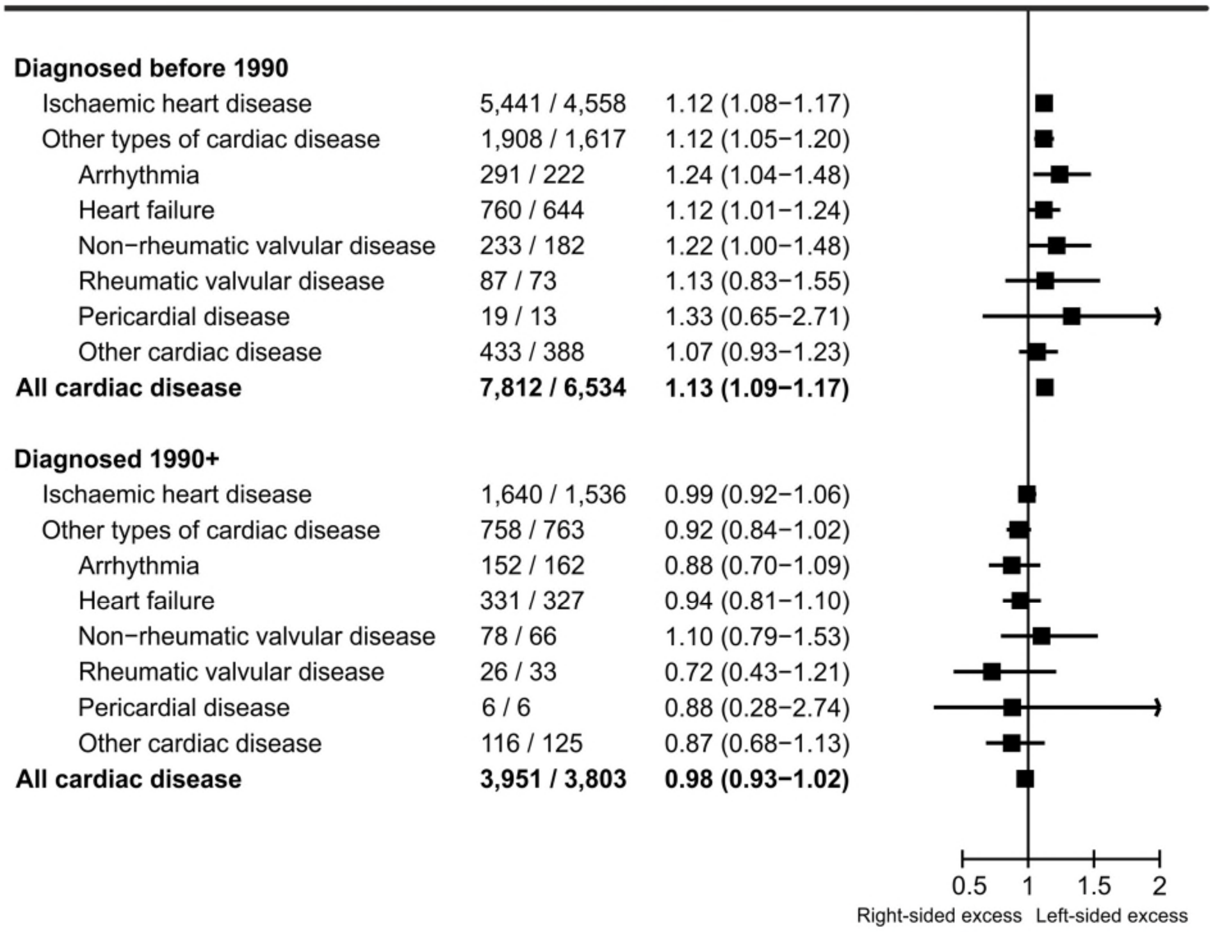

Figure S6. Irradiated women with left-sided breast cancer versus irradiated women with right-sided breast cancer: Cardiac mortality rate ratios, by calendar year of breast cancer diagnosis and certified cause of death.

See Table S2 for definitions of categories. Rate ratios estimated by Poisson regression with stratification by time since breast cancer diagnosis, age at breast cancer diagnosis, calendar year of breast cancer diagnosis, and country. Numbers of deaths from individual disease categories do not sum to totals as information on type of heart disease was unavailable from Surveillance, Epidemiology and End Results (SEER) for years 2000-2008, and was available only as two categories (ischaemic heart disease and other types of cardiac disease) from Ontario cancer registry.

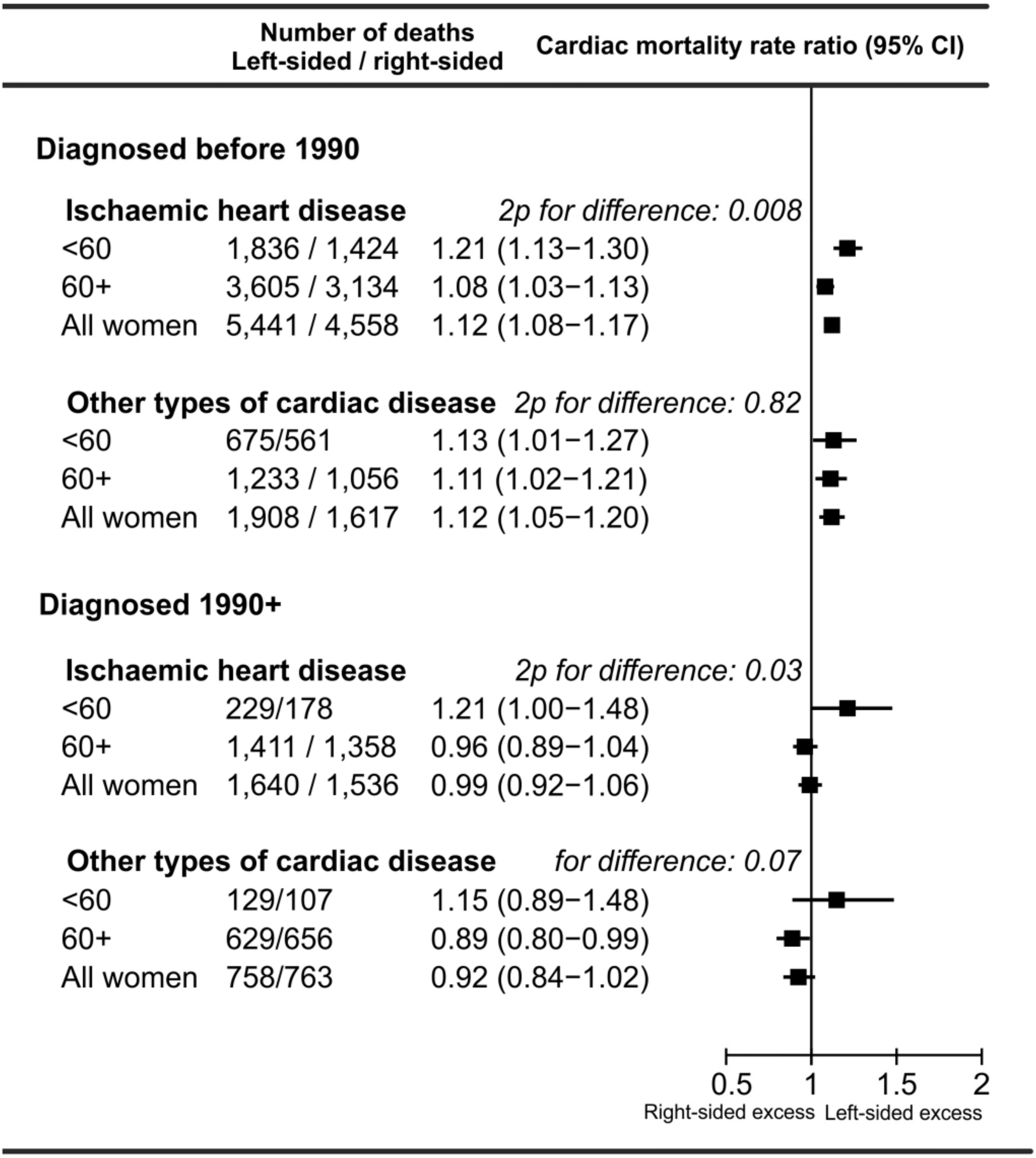

Figure S7. Irradiated women with left-sided breast cancer versus irradiated women with right-sided breast cancer: Cardiac mortality rate ratios, by age at diagnosis and whether the women were diagnosed with ischaemic heart disease.

Rate ratios estimated by Poisson regression with stratification by time since breast cancer diagnosis, age at breast cancer diagnosis, calendar year of breast cancer diagnosis, and country. Information on type of heart disease was unavailable from Surveillance, Epidemiology and End Results (SEER) for years 2000-2008.

Table S6. Breast cancer radiotherapy: Studies reporting cardiac risk according to age irradiated*

	Median age at RT (yr)	Design	Dosimetry	No. cardiac events after RT		Results according to age	
				All	Age <40	Cardiac risk	Heart dose (Gy)
Present study	57	Cohort<br>Left vs right	None	22,100<br>All time periods	340	**Cardiac mortality RR L vs R <1990**<br>Age <40 1.46 (1.15-1.86)<br>Age 40-49 1.19 (1.07-1.31)<br>Age 50-59 1.20 (1.12-1.28)<br>Age 60-69 1.09 (1.03-1.15)<br>Age 70-79 1.08 (1.02-1.15)<br>*p trend 0.003*	NS
Darby 2005	NS	Cohort<br>Left vs right	None	894	231	**Heart disease mortality ratio LvR**<br>Age 20-49 1.54 (1.08-2.19)<br>Age 50-59 1.53 (1.19-2.16)<br>Age 60-69 1.40 (1.15-1.70)<br>Age 70-79 1.28 (0.87-1.90)<br>*2p trend 0.40*	NS
McGale 2011	NS	Cohort<br>Left vs right	None	4291	104	**Heart disease incidence ratio LvR**<br>Age <60 1.12 (1.01-1.24)<br>Age 60-79 1.06 (0.98-1.15)<br>*p difference 0.4*	NS
Darby 2013	55	Case control	Individual	963	20	**Increase major coronary event rate per Gy**<br>Age 20-39 -1.5 (<25.3-616)<br>Age 40-49 6.3 (-2.0-41.3)<br>Age 50-59 7.1 (0.4-22.2)<br>Age 60-69 7.8 (1.7-19.7)<br>Age 70-74 9.7 (-2.9-116)<br>*p heterogeneity 0.99*	Age 20-39 4.7<br>Age 40-49 4.9<br>Age 50-59 5.1<br>Age 60-69 4.8<br>Age 70-74 4.9
EBCTCG 2017	56	Meta-analysis randomised trials	Trial-based	1253	14	**Cardiac mortality RR RT vs not**<br>Age <50 1.53 (1.07-2.19)<br>Age 50-59 1.27 (1.00-1.61)<br>Age 60+ 1.28 (1.10-1.48)<br>*p trend 0.5*	Age <50: 6.4<br>Age 50-59 6.2<br>Age 60+ 6.3
Jacobse 2019	50	Case control	Individual	183	9	**Excess rate ratio per Gy**<br>Age <45 24.2 (4.4-82.3)<br>Age 45-49 11.1(1.2-40.1)<br>Age 50-70 2.5 (-1.4-11.9)<br>*p trend 0.07*	NS
Paszat 1998	~60	Cohort<br>Left vs right	None	703	NS	**Myocardial infarction death RR LvR**<br>Age <60 1.98 (1.31-2.97)<br>Age ≥60 1.17 (0.95-1.44)<br>*P not specified*	NS

*Studies are ordered according to the number of cardiac events in women irradiated when aged <40 years
Abbreviations; vs=versus; RR=rate ratio; RT=radiotherapy; NS=Not specified; yr=years;Gy=gray

References for Table S6

Darby SC, McGale P, Taylor CW, Peto R. Long-term mortality from heart disease and lung cancer after radiotherapy for early breast cancer: prospective cohort study of about 300 000 women in US SEER cancer registries. Lancet Oncol 2005;6:557-565.

McGale P, Darby SC, Hall P, et al. Incidence of heart disease in 35,000 women treated with radiotherapy for breast cancer in Denmark and Sweden. Radioth Oncol 2011;100:167-175.

Darby SC, Ewertz M, McGale P, et al. Risk of ischemic heart disease in women irradiated for breast cancer. New England Journal of Medicine 2013;368:987-998.

Early Breast Cancer Trialists' Collaborative Group. Estimating the risks of breast cancer radiotherapy: Evidence from modern radiation doses to the lungs and heart and from previous randomised trials. J Clin Oncol 2017;35:1641-9.

Jacobse JN, Duane FK, Boekel NB, et al. Radiation dose-response for risk of myocardial infarction in breast cancer survivors. Int J Radiat Oncol Biol Phys 2019; 103: 595-604.

Paszat LF, Mackillop WJ, Groome PA, Boyd C, Schulze K, Holowaty E. Mortality from myocardial infarction after adjuvant radiotherapy for breast cancer in the Surveillance Epidemiology and End-Results Cancer Registries. J Clin Oncol 1998; 16: 2625-31.

Table S7. Hodgkin lymphoma radiotherapy: Studies reporting cardiac risk according to age irradiated*

	Median age at RT (yr)	Design	Dosimetry	No. cardiac events after RT		Results according to age	
				All	Age <40	Cardiac risk	Heart dose (Gy)
Van Nimwegen 2016	32	Case control study	Individual	325	251	**Excess relative risk incident coronary heart disease per Gy**<br>Age <27.5 20% (5.4-70.5)<br>Age 27.5-36.4 8.8% (2.6-22.9)<br>Age 36.5-50.9 4.2% (0.6-11.1)<br>*p interaction 0.15*	NS
Hancock 1993	29	Cohort	Individual mediastinal dose (not heart)	49	28	**Myocardial infarction death RR**<br>Age <20 44.1 (17.8-91.0)<br>Age 20-29 7.3 (3.4-13.8)<br>Age 30-39 5.1 (2.9-7.4)<br>Age 40-49 3.0 (1.4-5.5)<br>Age ≥50 1.8 (1.0-3.0)<br>*p trend <0.0001*	**Mediastinal dose**<br>Age <10 21.5<br>Age 10-19 37.3<br>Age 20-29 40.5<br>Age 30-39 38.8<br>Age 40-49 36.7<br>Age ≥50 28.7
Boivin 1992	NS	Case control	None	124	23	**Relative risk of acute myocardial infarction death**<br>Age 0-39 1.95 (0.25-15.35)<br>Age 40-59 1.44 (0.49-4.25)<br>Age 60+ 3.78 (1.12-12.80)<br>*P heterogeneity 0.64*	NS

*Studies are ordered according to the number of cardiac events in women irradiated when aged <40 years
Abbreviations; RR=rate ratio; RT=radiotherapy; NS=Not specified; yr: years; Gy: gray

References for Table S7

Van Nimwegen FA, Schaapveld M, Cutter DJ, et al. Radiation dose-response relationship for risk of coronary heart disease in survivors of Hodgkin lymphoma. J Clin Oncol 2016; 34: 235-43.

Hancock SL, Donaldson SS, Hoppe RT. Cardiac disease following treatment of Hodgkin's disease in children and adolescents. J Clin Oncol 1993; 11: 1208-15.

Boivin J-F, Hutchison GB, Lubin JH, Mauch P. Coronary artery disease mortality in patients treatment for Hodgkins disease. Cancer 1992; 69: 1241-7.
